# Distinctive genetic structure and selection patterns in *Plasmodium vivax* from South Asia and East Africa

**DOI:** 10.1038/s41467-021-23422-3

**Published:** 2021-05-26

**Authors:** Ernest Diez Benavente, Emilia Manko, Jody Phelan, Monica Campos, Debbie Nolder, Diana Fernandez, Gabriel Velez-Tobon, Alberto Tobón Castaño, Jamille G. Dombrowski, Claudio R. F. Marinho, Anna Caroline C. Aguiar, Dhelio Batista Pereira, Kanlaya Sriprawat, Francois Nosten, Robert Moon, Colin J. Sutherland, Susana Campino, Taane G. Clark

**Affiliations:** 1 Faculty of Infectious & Tropical Diseases, London School of Hygiene & Tropical Medicine, London, UK; 2 Public Health England Malaria Reference Laboratory, London School of Hygiene & Tropical Medicine, London, UK; 3 Grupo Malaria, Facultad de Medicina, Universidad de Antioquia, Antioquia, Colombia; 4 Department of Parasitology, Institute of Biomedical Sciences, University of São Paulo, São Paulo, Brazil; 5 Research Center for Tropical Medicine of Rondonia, Porto Velho, Brazil; 6 Shoklo Malaria Research Unit, Mahidol-Oxford Tropical Medicine Research Unit, Faculty of Tropical Medicine, Mahidol University, Mae Sot, Tak, Thailand; 7 Centre for Tropical Medicine and Global Health, Nuffield Department of Clinical Medicine Research Building, University of Oxford Old Road Campus, Oxford, UK; 8 Faculty of Epidemiology and Population Health, London School of Hygiene & Tropical Medicine, London, UK

**Keywords:** Genetic variation, Infection

## Abstract

Despite the high burden of *Plasmodium vivax* malaria in South Asian countries, the genetic diversity of circulating parasite populations is not well described. Determinants of antimalarial drug susceptibility for *P. vivax* in the region have not been characterised. Our genomic analysis of global *P. vivax* (*n* = 558) establishes South Asian isolates (*n* = 92) as a distinct subpopulation, which shares ancestry with some East African and South East Asian parasites. Signals of positive selection are linked to drug resistance-associated loci including *pvkelch10, pvmrp1, pvdhfr* and *pvdhps*, and two loci linked to *P. vivax* invasion of reticulocytes, *pvrbp1a* and *pvrbp1b*. Significant identity-by-descent was found in extended chromosome regions common to *P. vivax* from India and Ethiopia, including the *pvdbp* gene associated with Duffy blood group binding. Our investigation provides new understanding of global *P. vivax* population structure and genomic diversity, and genetic evidence of recent directional selection in this important human pathogen.

## Abstract (web summary)

The genetic diversity of *Plasmodium vivax* strains in South Asia isn’t well described. Here, the authors sequence *P. vivax* from returning UK travelers and establish South Asian isolates as subpopulation distinct from East African and South East Asian isolates.

## Introduction

*Plasmodium vivax* is now the leading cause of human malaria outside of sub-Saharan Africa^1^, where intensive disease-control interventions prioritising the control of *P. falciparum*, have had less impact on reducing the transmission of *P. vivax*^2^. Despite an estimated overall reduction of 61.0% in the burden of vivax malaria worldwide since 2000, there were 6.4 million cases reported in 2019^1^. Some countries have seen recent worrying plateauing in rates of case decline, or even increases (e.g., Papua New Guinea, Sudan, Yemen), partly associated with regions affected by political and economic instability, making the support of effective malaria control strategies difficult^2^. Further, drug resistance is potentially hindering malaria elimination. Resistance of *P. vivax* to chloroquine was first reported in 2003 in Papua New Guinea, and is now present in many *P. vivax* endemic countries^3^. As chloroquine susceptibility falls in *P. vivax* from these regions, the rates of treatment failure and relapse are increasing^1^. *P. vivax* has also developed resistance to the anti-folate drug combination sulfadoxine-pyrimethamine (SP), and possibly other antimalarial drugs such as mefloquine, used for *P. falciparum* where the two species are sympatric. As a result, artemisinin-based combination therapy (ACT) is now recommended for treating vivax malaria^1,4^. Other control measures which are traditionally effective against other *Plasmodium* parasites (e.g., *P. falciparum*) have proven to be less effective against *P. vivax*^5^ due to its ability to form dormant liver-stage parasites^6^. These hypnozoites cause delayed blood-stage relapses, but can be cleared from the liver with primaquine or tafenoquine treatments, although both regimens can cause severe haemolysis in individuals with G6PD deficiency^7^.

Countries in South Asia, including India, Afghanistan and Pakistan, account for nearly two-thirds of the total estimated *P. vivax* malaria cases globally, but there is little understanding of the parasite ancestry, genomic diversity and underlying factors governing transmission and drug resistance. Similarly, Eastern Africa accounts for a further 8% of the global cases^1^, but only in southern Ethiopia has genomic diversity been studied^8^. This contrasts with sequencing studies in South East Asia and South America, where genome-wide analyses have provided evidence of clustering of *P. vivax* in geographically distinct subpopulations, and for selective pressure on genetic regions encompassing some *P. falciparum* orthologous drug-resistance loci, including *pvdhps* and *pvdhfr* genes linked to SP^9–11^. Applying such population genetic approaches to identify determinants of drug resistance is important, because the understanding of biological susceptibility mechanisms including frontline chloroquine is poor; in part, hampered by the inability to long-term culture *P. vivax* parasites in the laboratory. Further, studies of in vivo drug efficacy are complicated by the difficulty of determining whether parasite reappearance comes from a new infection, recrudescence after treatment, or from relapse due to hypnozoite activation.

Whole-genome sequencing analysis has provided insights into *P. vivax* populations approaching elimination, which typically undergo a transition from high recombination rates and widespread intermixing, to clonal expansions with reduced genetic diversity^12^. Tracking this evolution of transmission patterns and inferring the geographical origin of isolates is aided by newly designed molecular barcodes^13^. Complementing these barcodes with South Asian and East African markers in putative drug-resistance genes will further assist infection control. Given the expansive *P. vivax* populations in the South Asia region are largely absent from population-based genomic studies, we have sequenced >118 *P. vivax* isolates in UK travellers with unambiguous travel histories returning from Bangladesh, India, Pakistan and Afghanistan. In general, travellers returning from malaria-endemic countries tend to have monoinfections, and provide an opportunity to investigate infections and drug efficacy in a potentially high-risk group^14^. The inclusion of these new South Asian parasite whole-genome sequences, together with new data generated from travellers to three East African countries (Eritrea, Sudan and Uganda) and previously published data, enables the largest study of *P. vivax* genomics to date (*n* = 558). By introducing data from little-studied geographic sources, our analysis provides expanded insights into the global ancestry, genomic diversity and population structure of *P. vivax*. We sought evidence that the substantial *P. vivax* populations of South Asia bear specific signals of selection in their genomes, and may harbour geographically distinct variants of drug susceptibility-associated loci and genes encoding red cell invasion proteins considered vaccine candidates. This knowledge can potentially inform the control of a disease with a high burden in this heavily populated region.

## Results

### Genome data and multi-clonality

A total of 388,933 high-quality SNPs were identified in the non-hypervariable regions in the genome of *P. vivax*. The final dataset comprised 558 isolates from 25 different countries (Supplementary Data 1), which were assigned into approximate regional groups: South Asia (*n* = 92; Afghanistan, Bangladesh, India, Sri Lanka, Pakistan; 89 returning travellers), East Africa (*n* = 50; Eritrea, Ethiopia, Madagascar, Sudan, Uganda; 25 returning travellers), South America (*n* = 146; Brazil, Colombia, Guyana, Peru, Mexico; 3 returning travellers), South East Asia (SEA; *n* = 186; Cambodia, China, Laos, Myanmar, Thailand, Vietnam), and Southern SEA (*n* = 84; Malaysia, Papua New Guinea, Indonesia, The Philippines; 1 returning traveller). A total of 118  *P. vivax* isolates from travellers entering the UK with unambiguous histories were sequenced de novo, covering five countries that have not previously undergone whole-genome sequencing.

Multi-clonality, as measured by within-sample diversity (F_WS_ metric >95%), was more common among SEA non-traveller isolates (69/186; 37.1%), than among non-traveller isolates from South America (24/146; 17.0%) or returning traveller isolates from both South Asia (15/88; 17.0%) and East Africa (3/25; 12.0%) (S1 Figure). This observation would suggest a lower proportion of superinfection and/or co-transmission of multiple strains in returning travellers, likely to simply reflect a restricted period of exposure to potentially infected *Anopheles* mosquitoes.

### *P. vivax* isolates from South Asia and East Africa are distinct subpopulations

**Fig. 1:**
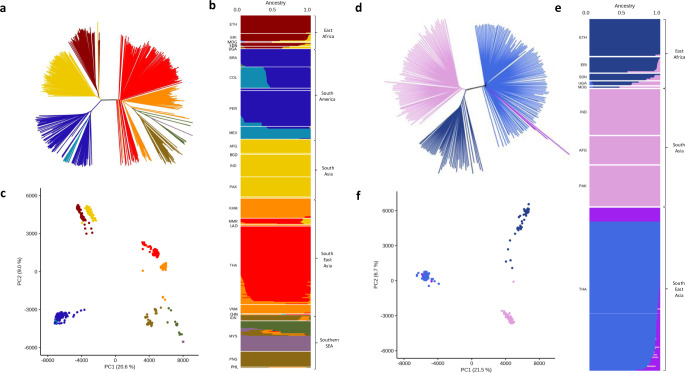
Population structure analysis using the whole set of 558 *P. vivax* isolates. Analysis of the whole set supports one unique non-differentiated subpopulation for the South Asian isolates. **a** Neighbour-Joining tree for the 558 isolates, constructed using a genetic distance based on 388,933 high-quality SNPs, and branches coloured based on (**b**); **b** ADMIXTURE prediction of a subpopulation (*K* = 9) visualised using a bar plot. **c** Principal Components Analysis (PCA) plot of the 558 isolates, with colours based on (**b**). **d** Neighbour-Joining tree for South Asia (India 36, Pakistan 32, Afghanistan 22), and East Africa (Ethiopia 29, Eritrea 12, Sudan 5, Uganda 3, Madagascar 1). Thailand isolates (128) were used as a representative population from South East Asia. Tree branches are coloured based on the assignment of the subpopulation from (**e**). **e** An ADMIXTURE bar plot illustrating the population structure (*K* = 4), highlighting a distinction between South Asian countries and Thailand, and East Africa and Thailand. The plot also highlights a degree of mixing between East African and South Asian populations. **f** PCA plot using the same data as in (**d**).

A SNP-based neighbour-joining tree revealed that South Asian and East African isolates occupy distinct positions as independent subpopulations within a global setting (Fig. 1). An admixture ancestral analysis suggested that these two subpopulations are amongst seven others, which represent isolates from South America (three subpopulations; Mexico, Peru, Colombia/Brazil), SEA (1), and Southern SEA (3). Further, some isolates in both the East African and the SEA subpopulations, especially Thailand and Myanmar, share this South Asian ancestry (Fig. 1). This population structure was also supported by principal components analysis (PCA) (Fig. 1), where analysis of South Asian isolates alone highlighted a further geographical separation of Indian isolates from Pakistan and Afghanistan’s subpopulations (S2 Figure); whilst in East African isolates, there was separation for isolates from travellers returning from Uganda compared to other East African countries. Furthermore, isolates from Eritrea and Sudan seemed to cluster together and mostly separated from Ethiopian isolates (S2 Figure).

### Analysis of IBD at the country level reveals similar regions of homology in India as in Ethiopia

Analysis of identity-by-descent (IBD) at the country level was performed in order to understand the chromosome-level structure in the subpopulations. The isolates obtained from returning travellers from India (median: 0.024), Pakistan (median: 0.013) and Afghanistan (median: 0.013) presented lower fractions of pairwise IBD overall than other isolates sampled in more geographically limited regions, such as Mexico (median: 0.23) or Malaysia (median: 0.33); but similar to other subpopulations in SEA, such as Thailand (median: 0.026) or Cambodia (median: 0.017) (S3 Figure). A genome-wide analysis of IBD fractions using sliding windows of 10 kbp revealed differences at the country level (S4 Figure and Supplementary Data 2). The regions with the highest IBD fractions across all three South Asia subpopulations included a segment of chromosome 10 encompassing the Myosin B protein (average IBD fraction: 0.074), the liver-specific protein 2 (LISP2) on chromosome 3 (0.064) and a cluster of  loci spanning the *pvmsp1* gene located on chromosome 7 (0.064) (Supplementary Data 2). IBD fraction signals specific to Indian isolates were identified in three regions at the end of chromosomes 2, 4 and 6, presenting a degree of overlap with the signals observed in Ethiopia; these regions included the *pvmrp1* gene (chr. 2; 150–160 kbp), a cluster of genes including PHIST proteins (chr. 4; 930–970 kbp) and the *pvdbp* gene and its neighbouring regions (chr. 6; 970–1010 kbp). Three Afghanistan-specific signals were observed, one on chromosome 1 (710–740 kbp) spanning six genes, and two on chromosome 11, including 630–660 kbp, spanning 7 genes (e.g., *pvpk5*), and 1050–1070 kbp spanning 6 genes (e.g., *pvsms1* and *pvsms2*). One signal specific to Pakistan corresponded to a region on chromosome 8 (280– 300 kbp) spanning five genes, including *pvgbp2* and *pvtmem147*. These regions could be candidates for inclusion in molecular barcodes for *P. vivax*.

### Population differentiation in putative drug resistance and mosquito-related genes

The SNPs underlying differences between subpopulations were identified using a fixation index (*F*_ST_) analysis. The *F*_ST_ distributions between putative drug resistance and other loci were different between SEA and South Asian or East African populations (Wilcoxon *P* < 0.012) (S5 Figure). The number of SNPs found to be highly differentiating (*F*_ST_ > 0.8) varied in number, with South Asia (*n* = 5) and SEA (*n* = 6) having much less than the other regions (Southern SEA 28; East Africa 43; South America 136) (Table 1 and Supplementary Data 3). There was high population differentiation in (putative) drug-resistance genes in SEA, particularly in *pvdmt1* (S310R and V217I) and *pvmrp1* (L1207I, V879 and T234M); for East Africa, South Asia and South America there was high differentiation in members of the *CRMP* gene family (*pvcrmp1*, *3* and *4*), implicated in mosquito transmission. Mutations in genes associated with mosquito stages of the parasite life cycle differentiated South Asian (*pvs28* and *pvs47)* and South American (*pvs47, pvs48/45*) populations, likely linked to differences in species of mosquito vectors, where *pvs47* has a role in parasite-mosquito interactions and its haplotypes present a marked geographical population structure^15^. Other loci with mutations with high *F*_ST_ in South Asia include the *PVP01_0514700* gene (*F*_ST_ = 0.95) which has an unknown function, and the protein disulfide isomerase *PDI8* (*F*_ST_ = 0.83). Similarly, East African-specific mutations occurred in the *pvclamp* gene linked to parasite motility, and *PVP01_1444400* (max. *F*_ST_ = 0.91) with unknown function; whereas, there was a cluster of mutations on chromosome 11 surrounding *PVP01_1129700* (max. *F*_ST_ = 0.99), which was South American-specific.

**Table 1:** SNPs with high *F*_ST_ ( > 0.8) that differentiate *P. vivax* isolates from South East Asia (SEA), East Africa and South Asia regions.

Region	Chr.	Position	Ref.	Alt	Effect*	AA change	Gene name	*F*_ST_**
SEA	2	155305	G	T	Non Syn	L1207I	*pvmrp1*	0.83
SEA	2	156287	C	A	Syn	V879	*pvmrp1*	0.82
SEA	2	158223	G	A	Non Syn	T234M	*pvmrp1*	0.81
SEA	14	1070933	A	T	Non Syn	S310R	*pvdmt1*	0.86
SEA	14	1071214	C	T	Non Syn	V217I	*pvdmt1*	0.90
South Asia	5	286241	A	T	Non Syn	I83F	*PDI8*	0.83
South Asia	5	618291	T	G	Non Syn	F165V	*PVP01_0514700*	0.95
South Asia	6	668360	G	T	Non Syn	T65K	*P28*	0.83
South Asia	12	323703	G	C	Non Syn	S57T	*P47*	0.81
South Asia	13	336960	C	A	Non Syn	T1455K	*CRMP3*	0.81
East Africa	4	308246	A	G	Intron	–	*CDPK1*	0.83
East Africa	4	401595	C	T	Syn	Y670	*ACS9*	0.81
East Africa	4	401608	T	C	Syn	L675	*ACS9*	0.85
East Africa	4	401622	C	T	Syn	N679	*ACS9*	0.90
East Africa	5	722792	T	G	Non Syn	I172L	*PVP01_0517200*	0.86
East Africa	5	1067476	C	T	Non Syn	E158K	*PVP01_0526300*	0.87
East Africa	5	1071079	T	G	Intron	–	*PVP01_0526400*	0.92
East Africa	6	646355	C	T	Non Syn	M236I	*CLAMP*	0.96
East Africa	6	646864	C	T	Non Syn	D67N	*CLAMP*	0.88
East Africa	6	661527	C	G	Non Syn	S273C	*RPN9*	0.85
East Africa	7	499047	T	G	Non Syn	I1234S	*CRMP1*	0.93
East Africa	7	507890	T	A	Syn	P582	*PVP01_0709900*	0.90
East Africa	7	509284	C	T	Non Syn	D118N	*PVP01_0709900*	0.96
East Africa	7	509311	A	G	Non Syn	Y109H	*PVP01_0709900*	0.95
East Africa	7	509312	G	A	Syn	L108	*PVP01_0709900*	0.95
East Africa	7	514538	A	G	Syn	L14	*PVP01_0710100*	0.91
East Africa	7	525785	T	C	Syn	A1270	*PVP01_0710500*	0.80
East Africa	7	556907	C	G	Non Syn	M709I	*PVP01_0711500*	0.89
East Africa	11	917226	A	T	Non Syn	F1138L	*PK4*	0.87
East Africa	11	1075544	C	G	Non Syn	S455R	*PVP01_1124700*	0.88
East Africa	12	1869662	T	C	Non Syn	V414A	*PVP01_1245200*	0.84
East Africa	13	334457	A	G	Non Syn	K621E	*CRMP3*	0.86
East Africa	13	334917	T	G	Non Syn	M774R	*CRMP3*	0.90
East Africa	13	610100	C	A	Non Syn	K841N	*PVP01_1313400*	0.89
East Africa	14	1296340	T	C	Syn	S205	*PPM5*	0.81
East Africa	14	1923223	G	A	Non Syn	P504S	*PVP01_1444400*	0.81
East Africa	14	1923997	T	G	Non Syn	M246L	*PVP01_1444400*	0.91
East Africa	14	1923998	C	G	Syn	T245	*PVP01_1444400*	0.91
East Africa	14	1924051	T	C	Non Syn	T228A	*PVP01_1444400*	0.91
East Africa	14	1924658	C	T	Syn	E25	*PVP01_1444400*	0.85

*AA* amino acid, *Non Syn non-synonymous mutation, *Syn* synonymous mutation, *Inter* intergenic region, *Start lost* start codon lost.

**Within Region vs. other isolates.

See Supplementary Data 3 for all regional comparisons.

### Identification of mutations in *P. vivax* drug-resistance candidate orthologues

With strong population-differentiating effects implicated in *Plasmodium* drug susceptibility loci in SEA, we identified non-synonymous (NS) mutations in *P. vivax* genes of interest and assessed their frequency across all regions: *pvcrt* (4 NS), *pvdhfr* (17), *pvdhps* (21), *pvkelch12* (*PVP01_1211100*; encoding a protein with 98% sequence identity with *pfK13* in the 306 amino acid propeller domain; 6 NS), *pvmdr1* (27), *pvmrp1* (41) and *pvpm4* (*plasmepsin4;* 3) (Supplementary Data 4). The majority of these mutations had low frequency across any region (60/119 mutations with maximum regional frequency <3%), including none in *pvcrt* or *pvkelch12*, suggestive of their lack of association with drug resistance in these populations. Of the four mutations found in *pvcrt*, two occurred in SEA (P38L Thailand; R121K Thailand/Cambodia), one in Mexico (E207Q), and one in Pakistan (P16L; *n* = 1). Similarly, for *pvkelch12*, two mutations outside the propeller domain were observed in South Asia (I6M, *n* = 2, India and Pakistan; N25I, *n* = 2, Pakistan and Thailand). No mutations in the *pvcrt* or *pvkelch12* genes were identified in East Africa. Finally, in the *pvpm4* gene, orthologous to piperaquine resistance-associated *pfpm4* in *P. falciparum*, only one mutation (I165V) was present across all regions including both South Asia (7.7%) and East Africa (24.5%), and two other mutations were present only in Papua New Guinea.

Mutations in *pvdhfr* (*PVP01_0526600*) and *pvdhps* (*PVP01_1429500*) genes in the anti-folate pathway are thought to contribute to SP drug resistance, although there are very few published studies that associate genotypes of these loci with anti-folate susceptibility phenotypes^16–18^. We observed several *pvdhfr* mutations (F57L, F57I, T61M, N117T) in SEA or Southern SEA, but these were absent from South Asia and East Africa (Table 2). An alternative amino acid substitution (N117S) was observed more prominently in South Asia (62.2%), although also present in South America (20.3%) and East Africa (6.3%). The S58R resistance allele was present across all regions, being fixed in SEA, common in East Africa (88.0%), but at lower frequency in South Asia (34.5%), where there was heterogeneity between countries (Pakistan and Afghanistan <19.8% vs. India 71.4%). Three mutations specific to South Asia were identified in *pvdhfr* (N50I, D243E and N273K) at low frequencies (range: 3.6–10.7%). For *pvdhps*, previously described mutations A553G and A383G were observed in high frequency in SEA (67.6% and 96.3%) and Southern SEA (54.1% and 68.9%), with lower frequencies in South Asia (11.0% and 13.3%) and East Africa (0% and 32.0%). Six mutations were unique to the South Asian region (M601I, D459A, M367L, F365L, I337V and E106D; < 7%) all present in low frequency across any of the three main countries (<13%). A further two mutations (L534I and E142G) were observed only in East Africa, with L534I exclusively in Uganda and E142G present across all four constituent countries. Based on in silico protein modelling, eight mutations in the PVDHFR (N50I, F57I/L, S/K58R, T61M, N117T/S) and four in the PVDHPS (A553G, G383A, S382C/A) are within a distance of 10 Å of the drug-binding site and could be expected to change the affinity of the drugs and proteins (Supplementary Data 5 and S6 Figure).

**Table 2:** Common mutations (allele frequency %) in putative drug-resistance genes.

Gene name	Position	Amino acid change	South Asia (*n* = 92)	East Africa (*n* = 50)	South America (*n* = 148)	SEA (*n* = 186)	South SEA (*n* = 84)
*pvdhfr*	1077510	N50I	7.1				
*pvdhfr*	1077530/2	F57I				**56.2**	
*pvdhfr*	1077530/2	F57L				4.3	**76.5**
*pvdhfr*	1077534	K58R			8.8		
*pvdhfr*	1077535	S58R	34.5	**88.0**	**67.9**	**100**	**62.2**
*pvdhfr*	1077543	T61M				**64.9**	36.5
*pvdhfr*	1077711	N117T			0.7	**64.1**	**72.6**
*pvdhfr*	1077711	N117S	**62.2**	6.3	20.3		9.5
*pvdhfr*	1078090	D243E	3.6				
*pvdhfr*	1078180	N273K	10.7				
*pvdhps*	1270256	M601I	6.6				
*pvdhps*	1270401	A553G	11.0		0.6	**67.6**	**54.1**
*pvdhps*	1270524	K512M				3.8	
*pvdhps*	1270683	D459A	3.3				
*pvdhps*	1270793	C422W					15.3
*pvdhps*	1270911	G383A	**86.7**	**68.0**	47.2	3.8	32.1
*pvdhps*	1270914	S382C			10.6		
*pvdhps*	1270915	S382A				21.1	
*pvdhps*	1270966	F365L	4.4				
*pvdhps*	1271444	M205I*		**72.0**	**59.2**	**100**	8.2
*pvdhps*	1271634	E142G*		**64.0**			
*pvdhps*	1271664	E132G*					38.8
*pvmdr1*	478789	L1449I	7.8				
*pvmdr1*	478955	K1393N	11.2	2.0		10.8	1.2
*pvmdr1*	479329	T1269S	10.1				
*pvmdr1*	479908	L1076F	2.2		**88.1**	33.5	1.2
*pvmdr1*	480207	F976Y	**97.8**	46.0	**89.9**	**68.7**	11.9
*pvmdr1*	480412	L908M			13.5		
*pvmdr1*	480552	A861E	4.4			3.8	
*pvmdr1*	480601	L845F	14.3			8.7	2.4
*pvmdr1*	480846	A763V				3.8	
*pvmdr1*	481042	S698G	**59.3**	38.0	**100**	0.5	
*pvmdr1*	481595	S513R	35.6	**72.0**	0.6	23.2	
*pvmdr1*	481636	D500N			17.3		
*pvmdr1*	481908	T409M	11.1				
*pvmdr1*	482473	V221L			18.9		
*pvmrp1*	153954	N1657S					30.1
*pvmrp1*	154065	I1620T					4.7
*pvmrp1*	154107	A1606D			7.5		
*pvmrp1*	154108	A1606T			7.6		
*pvmrp1*	154168	H1586Y	9.3		28.9	3.8	
*pvmrp1*	154216	D1570Y					3.5
*pvmrp1*	154294	V1544I			8.5		
*pvmrp1*	154350	T1525I			6.3		
*pvmrp1*	154492	I1478V	20.3	23.5	15.4		
*pvmrp1*	154668	G1419A	23.4	16.7	33.8		
*pvmrp1*	154747	D1393Y	18.3	4.9		0.5	
*pvmrp1*	154831	L1365F					3.5
*pvmrp1*	154843	L1361F	3.0			0.5	
*pvmrp1*	155080	L1282I			9.6		
*pvmrp1*	155305	L1207I				**85.4**	
*pvmrp1*	155871	C1018Y			6.1		
*pvmrp1*	156208	E906Q	42.6	48.6	24.8	4.3	9.4
*pvmrp1*	157300	K542E	14.1	2.5	1.4		
*pvmrp1*	158148	R259T*	39.3	**50.0**	13.3	0.5	
*pvmrp1*	158223	T234M				**84.0**	1.2
*pvpm4*	1802108	I165V	7.7	24.5	**66.3**	**56.2**	22.6

Allele frequency bolded if >50%; South Asia (Afghanistan, Bangladesh, India, Sri Lanka, Pakistan); East Africa (Eritrea, Ethiopia, Madagascar), South America (Brazil, Colombia, Guyana, Peru, Mexico); SEA (Cambodia, China, Laos, Myanmar, Thailand, Vietnam); Southern SEA (Malaysia, Papua New Guinea, Indonesia, The Philippines); * in the HPPK-coding part of the bifunctional gene.

*Pvmdr1* F1076L and Y976F mutations have been implicated in reduced chloroquine susceptibility, and F1076L was observed with high frequency across all regions (>66%; East Africa 100%, South Asia 97.8%) with the exception of South America (11.9%). The Y976F mutation was at high frequency in Southern SEA (88.1%) and also present in East Africa (54.0%) and SEA (32.3%), and at lower frequency in South Asia (2.2%) and South America (10.1%). Two mutations (S698G, S513R) were found at higher frequency in South Asia and East Africa (>35%) than in SEA and Southern SEA (< 1%), with S698G fixed in South America (100%). The N1657S mutation is found only in Malaysia. Eight mutations were observed in South Asia only, but only three (L1449I, T1269S, T409M) were common (>7%) (Table 2).

In the candidate *pvmrp1* gene, the high frequency of L1207I and T234M mutations exclusively in SEA (> 83%) coincided with the high population differentiation *F*_ST_ results (Table 1). Several mutations (I1478V, G1419A, D1393Y, E906Q, K542E, and R259T) were more frequent in isolates from South Asia and Africa, compared to Southern SEA and SEA. The I1478V, D1393Y, E906Q and R259T mutations were present in modest frequency in South American isolates (range: 4.9–48.6%). The most frequent mutations in East Africa (Ethiopia 51.9%) and South Asia (India ~45%), included R259T and E906Q with relatively low frequency (<10%) in both Southern SEA and SEA. Mutation D1393Y was found predominantly in South Asian isolates (18.3%), and G1419A was found in moderate frequency across South America (33.8%), South Asia (23.4%) and East Africa (16.7%) (Table 2). The comparison of orthologous amino acid positions (*pfmrp1* 1390 is *pvmrp1* 1298; *pfmrp1* 1466 is *pvmrp1* 1374) revealed the proximity of *pvmrp1* mutations (e.g., D1393Y, G1419A) to established drug-resistance phenotype causing mutations in *P. falciparum* in the vicinity of the 2^nd^ ABC transporter domain (S7 Figure). This relationship highlights the potential role of the *pvmrp1* mutations in driving drug resistance in *P. vivax* in an orthologous manner to the one observed in *P. falciparum*, whose function could be tested in in vitro assays using a *P. knowlesi* model^19^.

### Regions under selection in South Asian and East African subpopulations

Given the potential for population differentiation in putative drug-resistance genes, such as *pvmrp1*, we sought through a genome-wide analysis to identify if these or other loci are involved in selective sweeps. Analysis of positive selection within subpopulations was assessed using the integrated haplotype score (*iHS*) at a country and region level, for populations with at least ten isolates. We found regions containing the *pvmrp1* in SEA, the antigenic and potential vaccine targets *pvmsp4/5* genes in SEA, South America and Papua New Guinea; as well as further regions upstream of the *pvdhfr* gene, a region surrounding the MSP7-like cluster of genes on chromosome 12 in South America, and a cluster of *PHIST* exported genes on chromosome 14 in Papua New Guinea (all *P* < 10^−4^) (Supplementary Data 6 and  7).

**Fig. 2:**
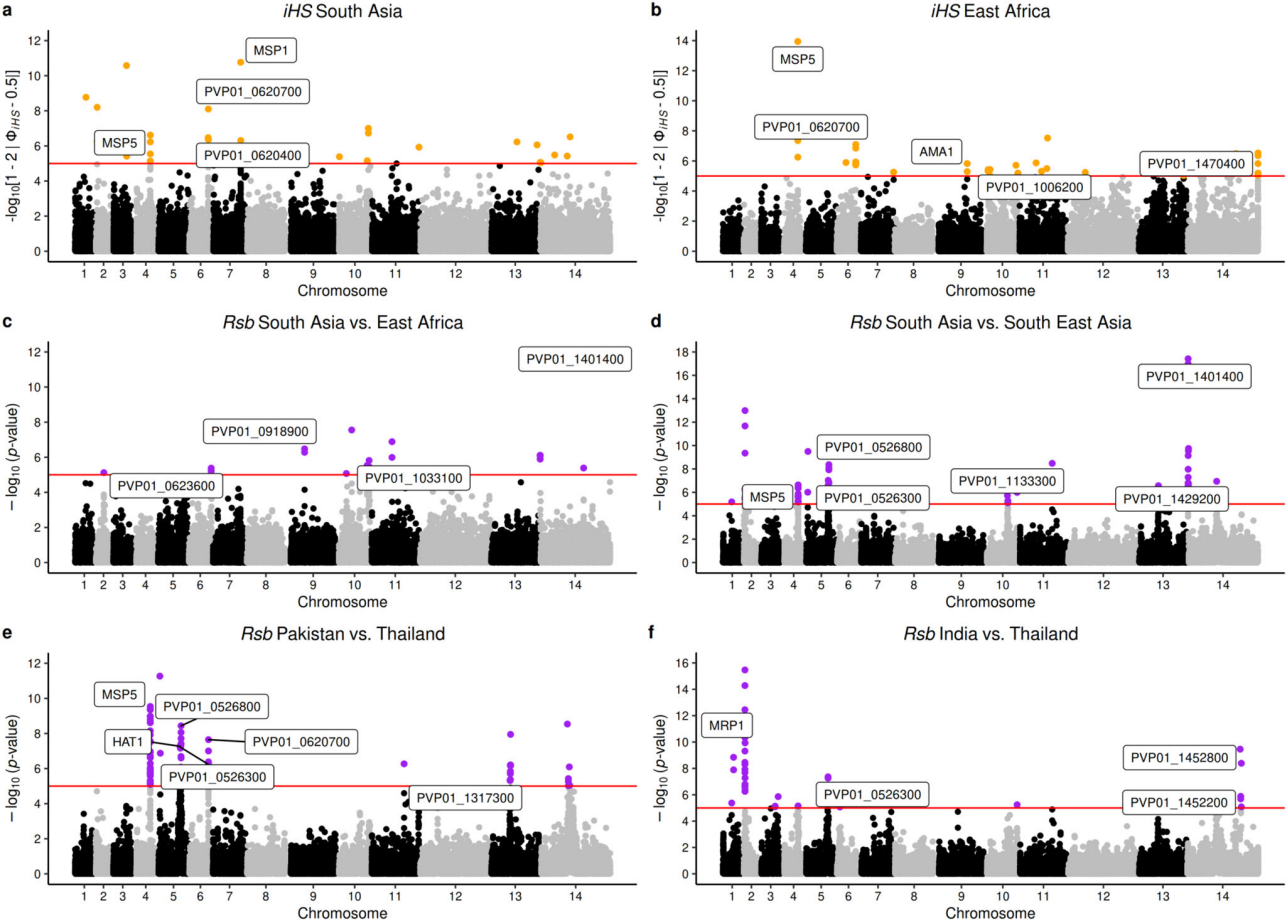
Evidence of selective sweeps. Manhattan plots showing the genome-wide results of the *iHS* analysis on *P. vivax* from South Asia and East Africa regions (**a, b**); *Rsb* analysis for *P. vivax* between geographical regions (**c**, **d**) and countries (**e**, **f**). Loci in critical regions (above red lines: orange points *iHS P* < 1 × 10^−5^; purple points *Rsb* P < 1 × 10^−5^; two-sided tests) are reported in Supplementary Data 6 and Supplementary Data 7.

For the South Asian subpopulations, ten chromosomal regions had multiple SNP hits (all *P* < 10^−4^), which included clusters surrounding *pvmrp1*, *pvcrmp2*, antigenic targets (*pvmsp1*, *pvmsp2, pvmsp4/5*), uncharacterised genes on chromosome 6 (*PVP01_0620400* to *_0621000*), reticulocyte-binding and invasion loci (*pvrbp1a/b*), the *pvdhfs* gene that encodes the bifunctional enzyme dihydrofolate synthase/folylpolyglutamate synthase (upstream of DHFR in the folate biosynthesis pathway), and uncharacterised exported proteins on chromosome 14 (*PVP01_1427400* to *_1427800*) (Fig. 2). In East Africa, selective sweeps were detected around  *pvmsp4/5*, the *pvabcg2* transporter and several *P. vivax* ApiAP2 transcription factors on chromosomes 12, 13 and 14 (Fig. 2). Country-level analyses reinforced the *pvmsp4/5* gene findings in Pakistan, Ethiopia and Eritrea (*P* < 1 × 10^−8^) (S8 Figure and Supplementary Data 6). In the isolates sourced from India, the strongest evidence for iHS sweeps were on chromosomes 11 (*PVP01_1126100* to *_1126500*) and 14 (*PVP01_1469900* to *_1470700*) which included tryptophan-rich protein-coding genes (e.g., *pvtrag23/24*; *P* = 1 × 10^−11^). Similarly, for the isolates from Afghanistan, the most significant SNPs were found in a cluster of exported proteins on chromosome 8 (*P* < 1 × 10^−10^), as well as a region surrounding the *pvkelch06* (*P* = 1 × 10^−6^), being orthologous to the artemisinin susceptibility-related *pfkelch10* gene^20,21^.

We identified evidence of geographically distinct signals of selection using the *Rsb* metric in 34 candidate loci with at least three significant SNPs (all *P* < 1 × 10^−5^) (Supplementary Data 7, Fig. 2 and S9 Figure). By comparing South Asia to SEA, the most significant hits were found proximal to drug-resistance candidate regions (*pvmrp1*, *pvdhfr* and *pvdhps*; *P* < 1 × 10^−7^), as well as a cluster including *pvmsp4/5*, and multiple genes on chromosomes 5 (*PVP01_0525800* to *_0526100*), 11 (*PVP01_1132900* to *_1133600*) and 14 (*PVP01_1401000* to *_1401900*). These results were supported by individual South Asian country comparisons with Thailand, including finding regions encompassing *pvdhfr* and *pvdhps* genes (all *P* < 1 × 10^−5^), and  *pvmrp1* (India *P* < 1 × 10^−15^). A comparison between South Asia and East Africa highlighted *pvdbp*, involved in red blood cell invasion, and *pvpk4* which is a drug target for inhibiting PK4 kinase activity with potency across species of malaria parasites. *P. vivax* isolates from Ethiopia displayed evidence of selection on regions of chromosomes 2 (including *pvmrp1*) and 10 (spanning *pvmsp8*), but with differential signals across South Asian population comparisons. Between country comparisons within South Asia (Supplementary Data 8, Fig. 2 and S10 Figure) using *Rsb* identified 71 regions (*P* < 1 × 10^−5^), and reinforced differences with isolates in India, including genetic regions encompassing *pvdbp*, *pvmrp1, pvmrp2*, sporozoite invasion-associated protein 2 (*pvsiap2*) and antigen UB05 (all *P* < 10^−11^). Only 17 differences occurred between Afghanistan and Pakistan, including *pvcrmp2* and a high density of exported proteins on chromosomes 6 and 8.

To study allele frequency distributions for individual genes across populations, our analysis focused on the 4826 loci that had at least five SNPs. Tajima’s D values were predominantly negative (median: East Africa −0.314, South America −0.453, South Asia −0.759, Southern SEA −0.887, SEA −1.692), being most negative in Thailand (median −1.545), consistent with a historical population expansion of *P. vivax* or directional selection (S11 Figure). There were strong correlations between Tajima’s D values within the same geographical region (Spearman’s correlations: Afghanistan/India/Pakistan 0.490–0.572; Thailand/Cambodia 0.507; Brazil/Colombia/Peru 0.250–0.308). Potential genes under balancing selection inferred by high Tajima’s D values (99.5 percentiles, Supplementary Data 9; values >2, Supplementary Data 10) included PHIST proteins (e.g., *PVP01_0525100*), antigenic targets (e.g., *pvCyRPA, pvmsp1, pvmsp5*) and conserved proteins of unknown function (e.g., *PVP01_0620700, PVP01_0203600*). From above, the pathogenesis-related *pvmsp5* locus also exhibited evidence of differential selection between populations, more broadly, reflecting observed complex haplotype structures of different strains circulating in different populations^8^.

Overall, the selection results reflect a picture of putative drug-resistance loci (e.g., *pvmrp1, pvdhfr* and *pvdhps*) with population-specific mutations within India, Afghanistan/Pakistan and Thailand, with signatures of directional selection. There was a modest correlation between selection (Rsb) and population differentiation (*F*_ST_) statistics in regional comparisons for coding SNPs (Spearman coefficient: median 0.3; min. 0.2 Southern SEA vs. East Africa or South America; max. 0.34 South Asia vs. East Africa), where high values for both metrics included mutations in *pvdhps, pvdbp, p28/47* mosquito-related and the CRMP family of genes (Supplementary Data 11).

## Discussion

Despite South Asian countries contributing collectively to more than half of the estimated *P. vivax* burden globally, the genomic diversity and population structure of parasites in this region is poorly understood. By evaluating samples from travellers returning to the UK with vivax malaria within the past 3 years, each with unambiguous travel histories to malaria-endemic countries, we have provided whole genome sequencing data for 132 *P. vivax* isolates. This increases the available global dataset to >700  isolates from at least 25 different countries. Analysis of the levels of multiplicity of infection revealed that the sequenced isolates from South Asian populations were less complex infections than has been reported for isolates from Thailand. This result may arise because travellers are exposed to fewer infective bites than people living permanently in a malaria-endemic region over long periods of time, and therefore on average are infected with fewer genotypes. By using either monoclonal or polyclonal samples with only a major dominant clone, differences in the multiplicity of infection linked to the source of parasite isolates (traveller vs. endemic populations) are unlikely to have had a major impact on our findings. However, we recognise that the exclusion of polyclonal infections disproportionately from endemic populations may have led to some underestimation of genetic diversity among these isolates compared to those from travellers. The lower multiplicity of infection in travellers (e.g., from South Asia) cannot be taken as an indication of lower transmission intensity in the source country. Further, the returning traveller samples were almost exclusively derived from passive sampling. Several studies have suggested that parasites derived from passively detected symptomatic infections have similar genotypes to actively detected asymptomatic infections^22^. These findings suggest that the development of clinical symptoms may be more attributable to host factors such as immunity and red blood cell polymorphisms, than to intrinsic parasite factors resulting in a higher multiplication rate. There are likely to be differences in demographic (e.g., age, sex), and health characteristics (e.g., the prevalence of comorbidities) in returning travellers compared to resident populations, which might increase either susceptibility to malaria infection or the risk of severe disease.

Population genetics analysis of the sequenced South Asian isolates provides evidence of an ancestral parasite subpopulation distinct from those in Africa, SEA and South America^8,10–12^. This subpopulation encompasses India, Afghanistan, Pakistan, Bangladesh and Sri Lanka, although isolates do share some features with parasite genomes from East Africa and SEA. This distinct characteristic is consistent with allopatric isolation by distance. Furthermore, at an intra-regional level, a degree of genetic separation was observed for isolates from India compared to Afghanistan and Pakistan, which are indistinguishable from each other, and an indication of parasite migration and gene flow between these two countries. In East Africa, despite the small sample size, a clear separation was observed for isolates from travellers returning from Uganda compared to other East African countries. Furthermore, isolates from Eritrea and Sudan seemed to cluster together and were clearly separated from Ethiopian isolates in the principal component analysis, although ADMIXTURE analysis did not reveal a separate ancestral population. This observation is consistent with recent reports of genetic diversity of *P. vivax* in Sudan using microsatellites^23^, but further studies of *P. vivax* in this region are warranted, given the recent history of cross-border refugee movements in the 20th and 21st centuries. *P. vivax* isolates from travellers to Guyana and The Philippines clustered near the South American and South East Asian populations as expected. The consistency of results at the geographic level combined with the observation that newly sequenced Ethiopian isolates clustered together with previously described Ethiopian sequences^8^ indicates that using returning travellers can be a robust approach to study difficult-to-access parasite populations. However, the typical lack of resolution of geographic information below national or, at best, provincial level, mean results should be backed up by future studies using systematically collected population samples from endemic communities in South Asia and East Africa.

Identity-by-descent (IBD) analysis was applied to further understand the structure and selection within parasite populations^24,25^. Analysis of the overall proportion of the genome that presented evidence of IBD revealed that isolates obtained from returning travellers displayed greater genetic diversity than in some highly related sampling populations, in particular Mexico and Malaysia. This result suggests that perhaps infections from returning travellers may be more diverse because they are not geographically defined by a given catchment area, whereas studies of endemic malaria tend to be geographically centred, and thus represent more coherent population samples. However, an alternative explanation might be that the parasite populations in Mexico and Malaysia are less diverse and highly related compared to all populations, because of lower transmission, leading to smaller effective population size. Our observed levels of IBD among traveller isolates from each country of interest were comparable to those of other populations such as Thailand or Cambodia, further supporting this alternative explanation. The genomic regions identified with a high proportion of IBD in different subpopulations included the liver-specific protein 2 (LISP2) on chromosome 3, a region on chromosome 10 including the Myosin B protein, and the *pvmsp1* gene region located on chromosome 7. A remarkable similarity was found between the regions of high IBD in India and Ethiopia, including a strong signal surrounding the *pvdbp* gene, the main invasion receptor that interacts with the human Duffy antigen to invade red blood cells. *P vivax* infections have now also been reported in Duffy-negative individuals, and *pvdbp* gene alterations, such as duplications have been suggested as a mechanism contributing to this adaptation^8,26,27^.

Population genetic analysis revealed genomic regions undergoing recent positive selection, including some encompassing loci linked to drug resistance such as the *pvmrp1, pvdhfr* and *pvdhps* genes^11^ in South Asia and other previously studied populations (e.g., Thailand), as well as in the *pvmsp4*/*5* regions on chromosome 4 for Ethiopia. Importantly, the analysis also revealed new regions which had not been previously reported as presenting with positive selection, including those surrounding the *pvrbp1a* and *pvrbp1b* genes and the folate pathway gene *pvdhfs* in South Asia. Furthermore, a region surrounding the *pvkelch10* gene was identified in Afghanistan as being under selection pressure, where this locus is an orthologue to the *pfkelch13* gene driving resistance to artemisinin in *P. falciparum*^28^*;* although no record of artemisinin resistance has been described in *P. vivax* currently, ACT regimens are now commonly used to treat *P. vivax* infections in many countries, reflecting guidance from the WHO^29^. Phenotypic information from in vivo ACT susceptibility studies linked to further evaluation of genetic variants at these loci is now needed to understand any selection acting upon the kelch propeller domain proteins of *P. vivax*.

The *pfmrp1* gene, encoding an ABC transporter transmembrane protein, is a potential multidrug-resistance candidate in *P. falciparum* with mutations associated with reduced susceptibility to SP (R1466K)^30^, chloroquine (F1390I)^31,32^, mefloquine (T1007M)^31^ and pyronaridine (H785N)^30–33^. Previous population genomic analyses of *P. vivax* populations in SEA and Oceania found evidence that the *pvmrp1* gene is under strong selection^10,11^, and our study provides evidence for this in South Asian isolates. Further, we found that *pvmrp1* contributes to IBD structure in other populations such as in Ethiopia. Previously unreported mutations were observed in South Asian populations, where *pvmrp1* exhibited a high frequency of two non-synonymous mutations (D1393Y and G1419A) compared to other regional subpopulations, supporting the strong positive selection signal. Furthermore, a comparison of the *pvmrp1* and *pfmrp1* sequence similarity revealed that non-synonymous mutations found in *pvmrp1* specific to the South Asia region, overlap with *pfmrp1* locations residing between the ABC transmembrane and the second ABC transporter domains. *Pfmrp1* mutations have been shown to confer resistance experimentally in *P. falciparum*^30,32^. In the absence of a long-term *P. vivax* in vitro culture system for validation, experimental strategies such as orthologue replacement transgenesis in *P. knowlesi*, in which *P. vivax* alleles of interest replace endogenous loci, can allow in vitro assessment of drug susceptibility for these variants^19^.

Overall, this study has provided analyses of *P. vivax* population structure and genomic diversity in three high burden South Asian countries, and further advanced our understanding of the genomic composition of *P. vivax* around the globe^9,10^. Despite some limitations of sampling from returning travellers, including reduced levels of the multiplicity of infection and therefore disconnection from transmission intensity levels in the origin populations, this approach has delivered valuable genome-wide data from otherwise uncharted *P. vivax* parasite populations. The evidence presented relating to positive selection evidence in the South Asian region for drug-resistance candidates such as *pvmrp1* is founded on previously established findings in other endemic regions^8,10–12^. The direct investigation of the phenotypes of gene variants in *P. vivax*^19^, and identification of potential genetic drivers of drug resistance using recent advancements in laboratory strategies such as orthologue replacement, is now possible.

## Methods

### Isolates and sequence data used for analysis

A total of 730 *P. vivax* isolates were included in the study, including previously publicly available data^8–10,12,13^ (*n* = 598) and newly sequenced (*n* = 132) isolates from imported UK cases with blood samples sent to the Public Health England Malaria Reference Laboratory. After quality control, the final dataset included 558 isolates from 25 countries spanning all the *P. vivax* endemic regions (Supplementary Data 1): South America (*n* = 146; Brazil (31), Colombia (34), Guyana (3), Peru (58), Mexico (20)), East Africa (*n* = 50; Eritrea (12), Ethiopia (29), Madagascar (1), Sudan (5), Uganda (3)), South Asia (*n* = 92; Afghanistan (22), Bangladesh (1), India (36), Sri Lanka (1), Pakistan (32)), South East Asia (SEA; n = 186; Cambodia (32), China (1), Laos (2), Myanmar (9), Thailand (128), Vietnam (14)) and the Western Pacific and southern South East Asia (Southern SEA; *n* = 84; Malaysia (49), Papua New Guinea (25), Indonesia (9), The Philippines (1)). These included both publicly available data (440/598; 73.6%) and newly sequenced samples (118/132; 89.4%). Sequencing of the newly generated isolates (*n* = 132) used DNA extracted from blood samples sourced from returning travellers to the UK from endemic areas between 2017 and 2018 and was performed on an Illumina HiSeq 4000 platform. The 132 samples were sourced from travellers to Afghanistan (*n* = 26), Bangladesh (*n* = 1), Eritrea (*n* = 11), Ethiopia (*n* = 6), Guyana (*n* = 3), India (*n* = 38), Pakistan (*n* = 35), The Philippines (*n* = 1), Sudan (*n* = 7) and Uganda (*n* = 4). For the isolates from India, country regional level data were available for 32 isolates, with 10 being from Mumbai, 8 from Gujarat, 7 from Goa and 7 from Punjab. The UK National Research Ethics Service (Ref: 18/LO/0738) and LSHTM Research Ethics Committee (Ref: 14710) provided approval for the project “Drug susceptibility and genetic diversity of imported malaria parasites from UK travellers”. Informed consent was obtained from all UK traveller study participants.

### Raw reads mapping and bam file correction

The Illumina sequencing reads were first filtered using *trimmomatic* (version 0.39) and the parameters: LEADING:3 TRAILING:3 SLIDINGWINDOW:4:20 MINLEN:36. Reads were then mapped to the *P. vivax* P01 (PvP01; version 1) reference genome^34^ using *bwa-mem* (version 0.7.12). The *samtools*^35^ (version 1.9) functions fixmate and markdup were applied to the resulting BAM files. The GATK’s BaseRecalibrator and ApplyBQSR functions were applied using a combined VCF file with SNPs identified in previous datasets as being high quality and presenting a minimum allele frequency (MAF) > 5%^11^. A set of 730 improved BAM files was obtained. Alignment metrics were calculated using the flagstats option within the samtools software.

### Variant discovery and quality control

The discovery of SNPs and indels was performed using GATK’s HaplotypeCaller (version 4.1.4.1) and followed recommended best practice^36^. GATK was run with default parameters and using the option -ERC GVCF. The GVCFs were validated using the GATK ValidateVariants function, then imported into a GenomicDB using the GATK function GenomicsDBImport, and a combined VCF was created using the GATK GenotypeGVCFs function. A total of 1,384,569 SNPs were identified. Variants in subtelomeric regions and internal *vir* genes regions were excluded. Subtelomeric regions in the 14 chromosomal sequences were identified by mapping previously published regions from *P vivax* Salvador I (PvSal1, version 1.0) into the PvP01 reference (Supplementary Data 12). Variants were then assigned a quality score using GATK’s Variant Quality Score Recalibration (VQSR). VariantRecalibrator was run using the previously mentioned dataset as a training set with a prior of 15.0. For SNPs we used the following parameters: -an QD -an FS -an SOR -an DP–maxGaussians 4 and --mq-cap-for-logit-jitter-transform 70. ApplyVQSR was then run using the parameter --truth-sensitivity-filter-level 99.0 to obtain a Variant Quality Score Log-Odds (VQSLOD). Variants with a VQSLOD score < 0, representing variants more likely to be false than true, were filtered out.

A total of 172 isolates with >40% of SNPs missing genotype data, as a result of poor sequencing coverage, were excluded from downstream analysis. The excluded isolates consisted of 158 (91.9%) publicly available (Peru 38, Brazil 23, Colombia 22, Mexico 19, Papua New Guinea 19, Cambodia 10, Thailand 9, Vietnam 7, Myanmar 6, Nicaragua 2, Panama 2, India 1) and 14 (8.1%) newly sequenced traveller samples (Afghanistan 3, Eritrea 3, India 2, Pakistan 2, Sudan 2, Bangladesh 1, Guyana 1). Source countries with excluded isolates had other samples that passed quality control, ensuring no loss in global representation. SNPs were then processed to replace the genotype call in the VCF for a mixed call whenever the secondary MAF was at least 20% in a given SNP on each individual sample. A subset of only biallelic SNPs was also obtained from this matrix. The final matrix consisted of 558 isolates (118 from travellers) and 388,933 high-quality SNPs. Variants were annotated using snpEff (version 4.1)^37^ with the following options: -no-downstream -no-upstream. A summary of the analysis pipeline applied is presented in S12 Figure.

### Multiplicity of infection

Multiplicity of infection (MOI) was assessed by grouping isolate data at a country level and calculating the *F*_WS_ score^38^, as well as at an individual isolate level using *estMOI*(version 2.0)^39^. Only SNPs in coding regions which were biallelic were used for the calculation of *F*_WS_. *F*_WS_ was calculated using the *moimix* (version 0.0.2.9001) package (https://github.com/bahlolab/moimix).

### Population genetics

A principal component analysis (PCA) was performed on the high-quality SNPs and isolates by calculating the pairwise genetic distance using the Manhattan method. A neighbour-joining tree was obtained to represent these similarities, and was generated using the *ape* package (version 5.4-1)^40^. A total of 135,954 high-quality SNPs with MAF > 1% were used to assess the population structure within the ADMIXTURE (version 1.3.0) package, where the most likely number of subpopulations (*K*) was obtained using cross-validation error ^41^. In order to assess the SNPs driving the allele frequency differences between populations, we identified high fixation index *F*_ST_^42^ values using the *PopGenome* library (version 2.7.5) in R. Isolate populations were compared at both a regional and country level.

### Positive and balancing selection and IBD analysis

Isolate populations at both country and regional level with >10 isolates were screened for positive selection using the *rehh* package (version 3.2.1) in R^43^. Isolates with *F*_WS_ < 95% were excluded from this analysis in order to use only monoclonal infections. *EstMOI* estimated that MOI was 1 for the majority of evaluable included isolates, with less than thirty displaying an MOI of 2, and for these, dominant clones had a relative abundance of at least 90% in every case. Both the integrated haplotype score (*iHS*)^44^ for within-population selection and *Rsb* for identification of selection between two populations^45^ were calculated. Calls obtained from the *P. cynomolgi* data (Accession number: DRR000476) were used as the ancestral states of the SNPs in our dataset^12^. Critical regions were identified using a 10 kbp sliding window including at least three SNPs with a *P* value <1 × 10^−4^ for *iHS* and a *P* value <1 × 10^−5^ for *Rsb*. Tajima’s D was calculated at a country level using the package *pegas* (version 0.14) in R for populations with >10 isolates and for genes with >5 SNPs. A negative Tajima’s D value may indicate population size expansion and/or purifying selection. Whilst a positive value may indicate a decrease in population size and/or balancing selection. Identity-by-descent (IBD) screening was also performed at a country level using the package *hmmIBD* (version 2.0.4)^24^ for populations with >10 isolates and excluding isolates with *F*_WS_ > 95%. Default parameters, including recombination rate, were used and based on *P. falciparum* estimates^24^. The proportion of pairwise comparisons for isolates presenting evidence of IBD was plotted by genome location using a sliding window of 10 kbp.

### Comparison of *pvmrp1* and *pfmrp1* genes

*P. vivax* orthologues of *P. falciparum* genes are based on high levels of identity and similarity in the encoded amino acid sequences^46^. The protein sequences for *pvmrp1* and *pfmrp1* genes were downloaded from PlasmoDB (https://plasmodb.org) with gene ID *PF3D7_0112200* for *pfmrp1* and *PVP01_0203000* for *pvmrp1,* and then aligned using the *mafft* protein aligner (https://mafft.cbrc.jp/alignment/software/). Protein domains predictions were also accessed through the PlasmoDB website.

### Protein structural modelling

Protein structures for DHFR and DHPS were downloaded from the Protein Data Bank (PDB) (https://www.rcsb.org/) (DHFR: 2BL9, DHPS: 5Z79). The structures were visualised and annotated using UCSC chimera. Distance from the ligand was calculated using the mCSM-lig webserver (http://biosig.unimelb.edu.au/mcsm_lig).

### Reporting summary

Further information on research design is available in the Nature Research Reporting Summary linked to this article.

## Supplementary information

Supplementary Information

Description of Additional Supplementary Files

Supplementary Data 1

Supplementary Data 2

Supplementary Data 3

Supplementary Data 4

Supplementary Data 5

Supplementary Data 6

Supplementary Data 7

Supplementary Data 8

Supplementary Data 9

Supplementary Data 10

Supplementary Data 11

Supplementary Data 12

Reporting Summary

## Data Availability

Protein structures for DHFR (2BL9) and DHPS (5Z79) were available from the PDB. All raw sequence data is available from the European Nucleotide Archive (www.ebi.ac.uk/ena; see Supplementary Data 1 for accession numbers). These data include samples from the LSHTM returning travellers (PRJEB44419) and the MalariaGEN *P. vivax* Genome Variation project (see ref. ^10^).

## Code availability

All scripts use open source software (see “Methods”).
