# Supplementary material for: Distinctive genetic structure and selection patterns in Plasmodium vivax from South Asia and East Africa
Source: Nat Commun. 2021 May 26;12:3160. doi: 10.1038/s41467-021-23422-3 (PMC8154914; doi:10.1038/s41467-021-23422-3)

Supplementary Information file for

Distinctive genetic structure and selection patterns in *Plasmodium vivax* from South Asia and East Africa

Ernest Diez Benavente [1,&], Emilia Manko [1,&], Jody Phelan [1], Monica Campos [1], Debbie Nolder [1,2], Diana Fernandez [3], Gabriel Velez-Tobon [3], Alberto Tobón Castaño [3], Jamille G. Dombrowski [4], Claudio R. F. Marinho [4], Anna Caroline C Aguiar [4], Dhelio Batista Pereira [5], Kanlaya Sriprawat [6], Francois Nosten [6,7], Robert Moon [1], Colin J. Sutherland [1,2], Susana Campino [1,*], Taane G. Clark [1,8,*]

[1] Faculty of Infectious & Tropical Diseases, London School of Hygiene & Tropical Medicine, London, United Kingdom

[2] Public Health England Malaria Reference Laboratory, London School of Hygiene & Tropical Medicine, London, United Kingdom

[3] Grupo Malaria, Facultad de Medicina, Universidad de Antioquia, Antioquia, Colombia

[4] Department of Parasitology, Institute of Biomedical Sciences, University of São Paulo, São Paulo, Brazil

[5] Research Center for Tropical Medicine of Rondonia, Porto Velho, Brazil

[6] Shoklo Malaria Research Unit, Mahidol-Oxford Tropical Medicine Research Unit, Faculty of Tropical Medicine, Mahidol University, Mae Sot, Tak, Thailand

[7] Centre for Tropical Medicine and Global Health, Nuffield Department of Clinical Medicine Research Building, University of Oxford Old Road Campus, Oxford, United Kingdom

[8] Faculty of Epidemiology and Population Health, London School of Hygiene and Tropical Medicine, London, United Kingdom

* Corresponding authors

E-mail: taane.clark@lshtm.ac.uk, Susana.campino@lshtm.ac.uk

[&] These authors are joint authors

S1 Figure. Multiplicity of infection. (top) Boxplot representing F_{WS} for *P. vivax* isolates grouped by country. Only countries with more than 2 isolates were included. All boxplots consist of boxes (median and interquartile range) and whiskers that extend to the most extreme data point which is no more than 1.5 times the interquartile range from the box. **(bottom)** F_{WS} cumulative plot highlighting a lower proportion of superinfections in South Asian and (non-Ethiopian) East African countries, compared to Thailand which has the highest proportion ($F_{WS} > 95\%$). The analysis includes Afghanistan (22), Pakistan (32), India (36), Sudan (5), Uganda (3), Eritrea (12), Ethiopia (29), Vietnam (14), Myanmar (9), Cambodia (32), Thailand (128), Indonesia (9), Papua New Guinea (25), Malaysia (49), Brazil (31), Colombia (34), Peru (58), and Mexico (20).

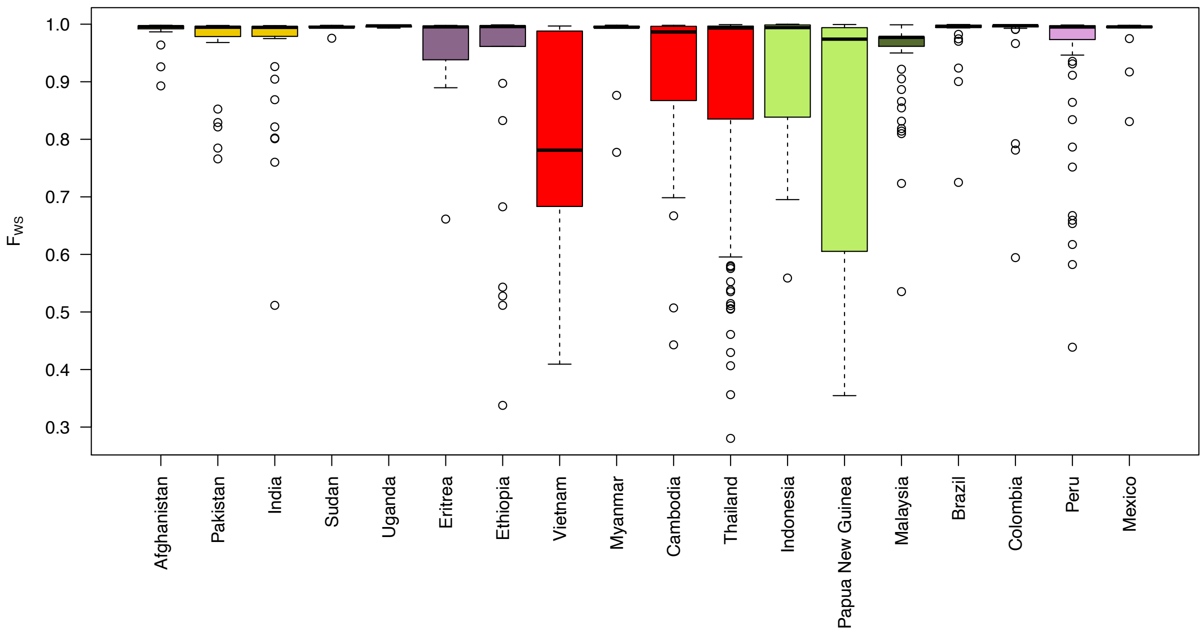

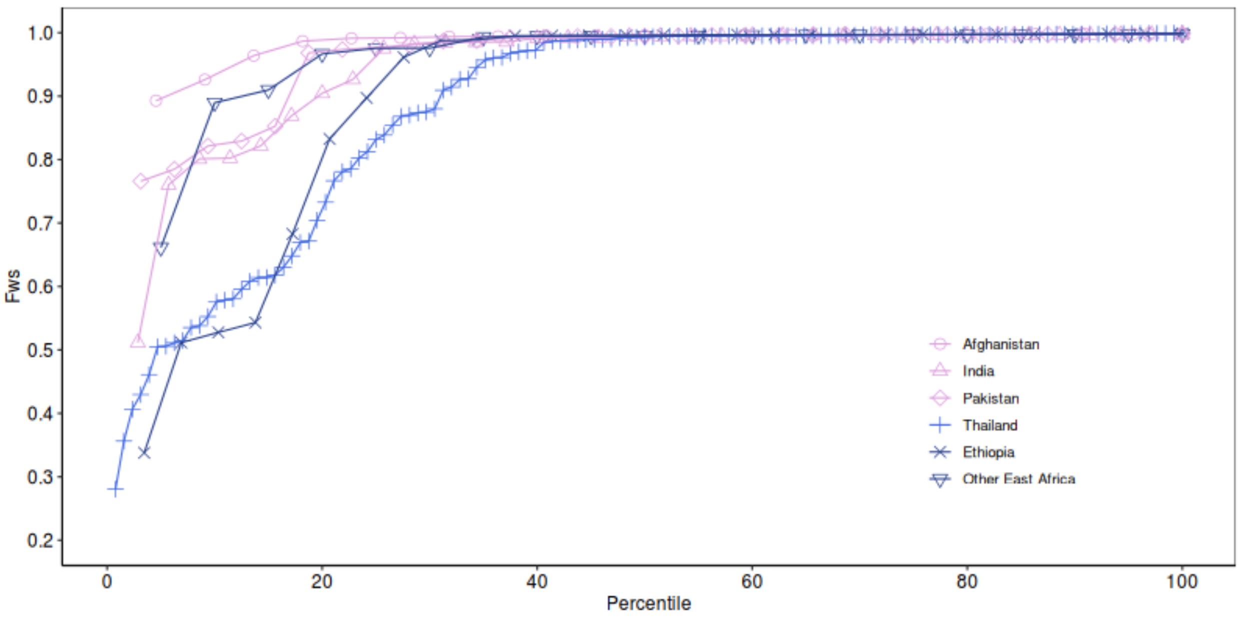

S2 Figure. Population structure of *P. vivax* isolates from South Asia and East Africa. (A) Principal Components Analysis (PCA) using the pairwise isolate distance (388,933 high-quality SNPs) for all populations, coloured by geographical region; **(B)** PCA plot for isolates from South Asia (India 35, Pakistan 32, Afghanistan 22) and East Africa (Ethiopia 29; Eritrea 12, Sudan 5, Uganda 3, Madagascar 1). Thailand isolates (144) were used as a representative population from South East Asia; coloured by geographical region; **(C)** PCA plot of South Asian isolates, coloured by country; **(D)** PCA plot of East African isolates, coloured by country.

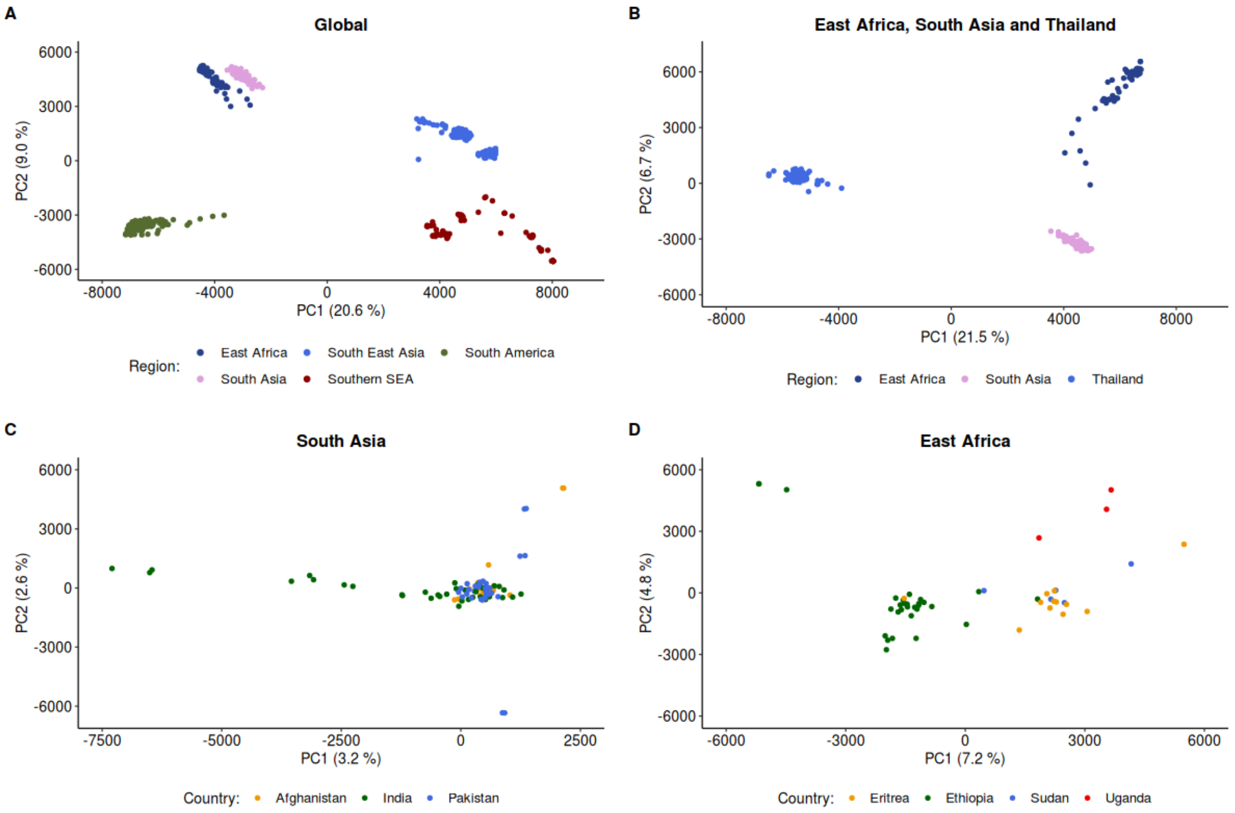

S3 Figure. Pairwise identity-by-descent (IBD)-fractions across country-level* subpopulations of *P. vivax* isolates. Populations with at least 10 isolates with $F_{WS} > 0.95$ are presented. All boxplots consist of boxes (median and interquartile range) and whiskers that extend to the most extreme data point which is no more than 1.5 times the interquartile range from the box. The analysis includes Afghanistan (20), Pakistan (27), India (28), Ethiopia(22), Cambodia (20), Thailand (84), Papua New Guinea (15), Malaysia (37), Brazil (28), Colombia (31), Peru (44), and Mexico (18).

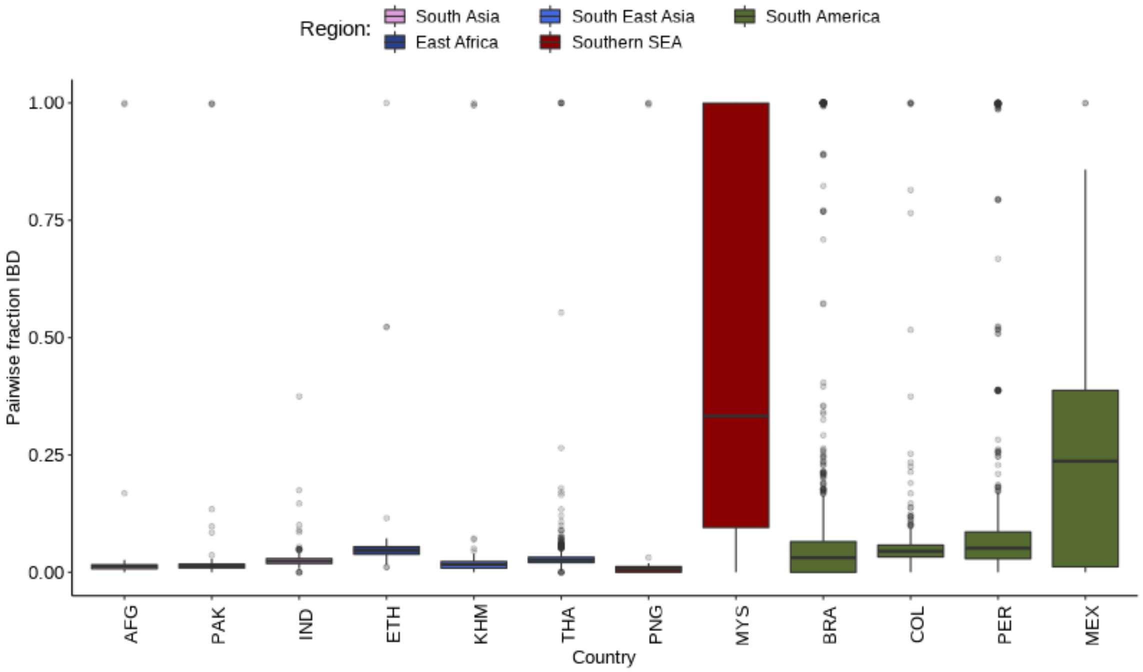

*AFG Afghanistan, PAK Pakistan, IND India, ETH Ethiopia, KHM Cambodia, THA Thailand, PNG Papua New Guinea, MYS Malaysia, BRA Brazil, COL Colombia, PER Peru, MEX Mexico

S4 Figure. Genome distribution of pairwise identity-by-descent (IBD) fractions across country-level subpopulations of *P. vivax* isolates. The top 1% of genomic regions for each country's parasite population are included in **S2 Table.**

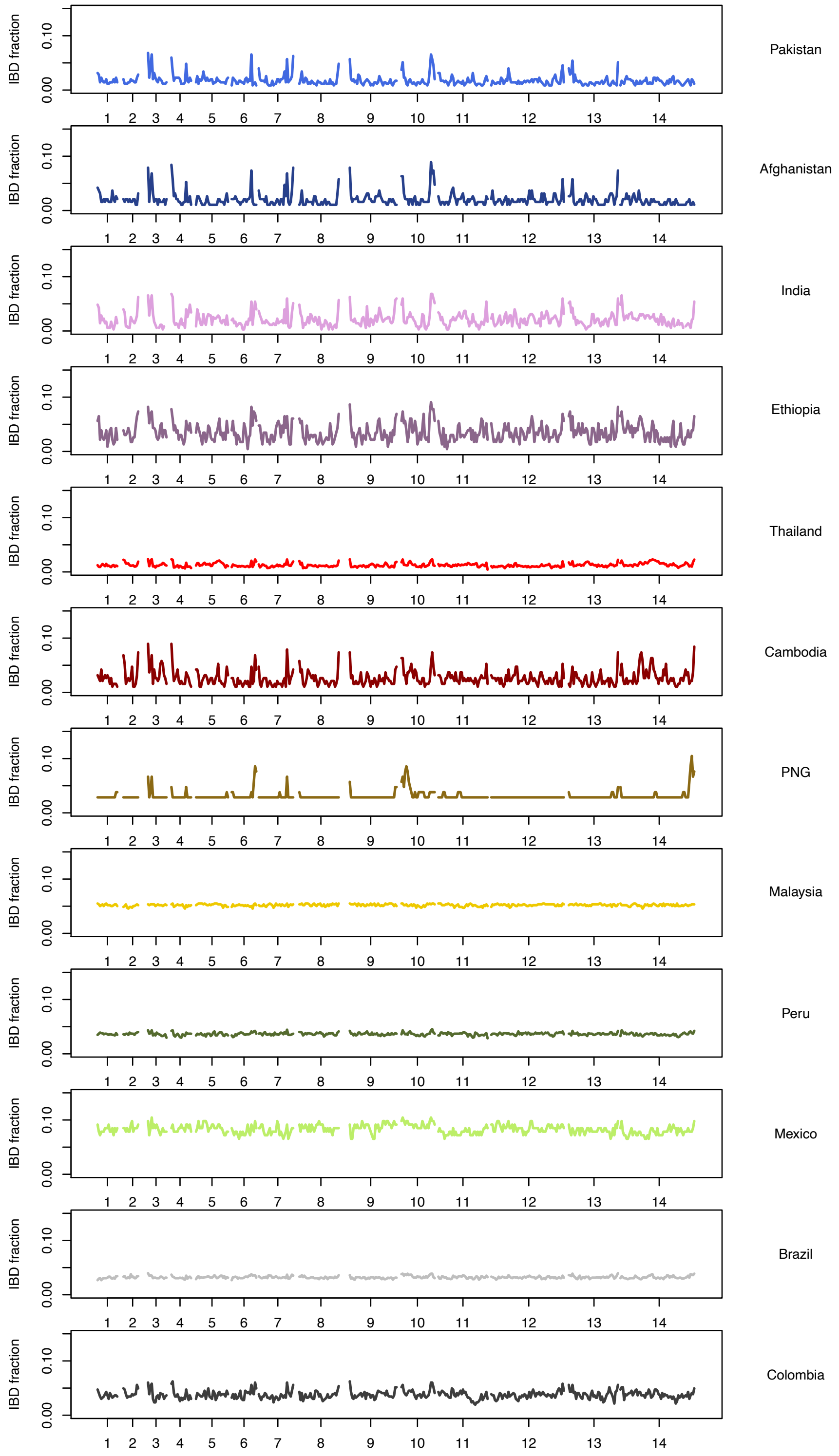

S5 Figure. Population Fixation F_{ST} distributions between (putative) drug resistance* and other loci across regional sub-groups.** All boxplots consist of boxes (median and interquartile range) and whiskers that extend to the most extreme data point which is no more than 1.5 times the interquartile range from the box. The analysis includes South Asia (92), East Africa (50), South East Asia (SEA, 186), Southern SEA (84), and South America (146).

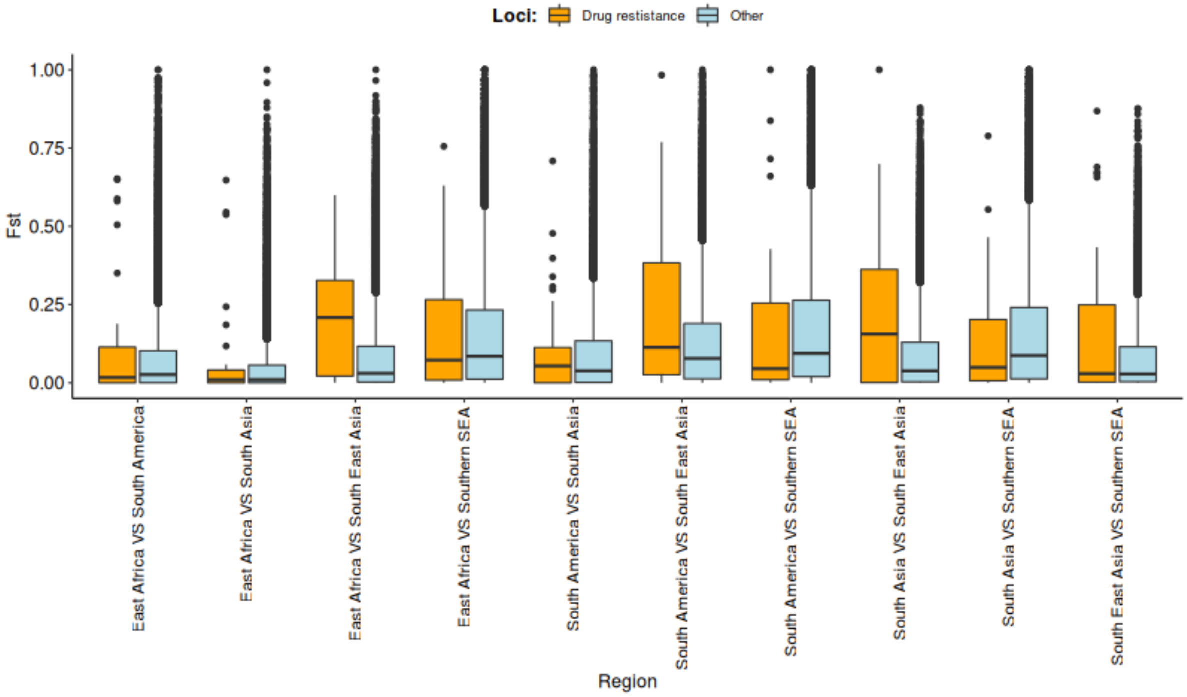

* *pvdhfr, pvdhps, pvmdr1, pvpm4, pvmrp1, pvcrt*; 42 SNPs; ** 43,903 SNPs; only the differences between South East Asia and South Asia (Wilcoxon P=0.011; two-sided) or East Africa (Wilcoxon P=0.0001; two-sided) were statistically significant

S6 Figure. Protein structures for PVDHFR and PVDHPS. Amino acid positions altered by SNPs are highlighted in blue. A high density of mutations is observed in residues in close proximity to the respective drug binding sites (**(A)** Pyrimethamine (*pvdhfr*); **(B)** 4-aminobenzoic acid (*pvdhps*)), which are highlighted in red. Mutations are labelled with the codon number and residue present in the PDB structure (**(A)** DHFR 2BL9; **(B)** DHPS 5Z79; https://www.rcsb.org).

(A) PVDHFR protein (target for pyrimethamine)

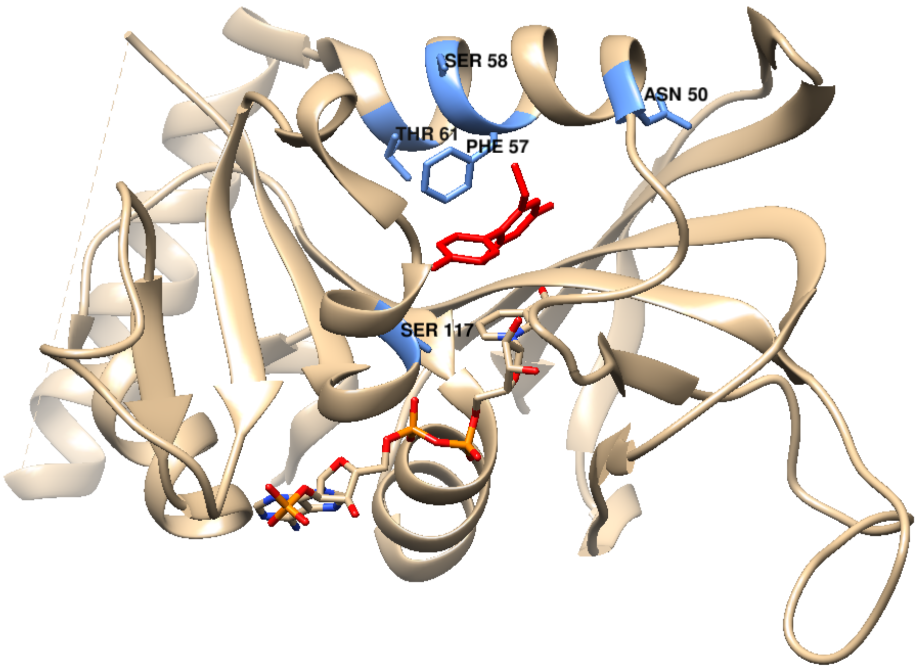

(B) PVDHPS protein (target for sulfadoxine)

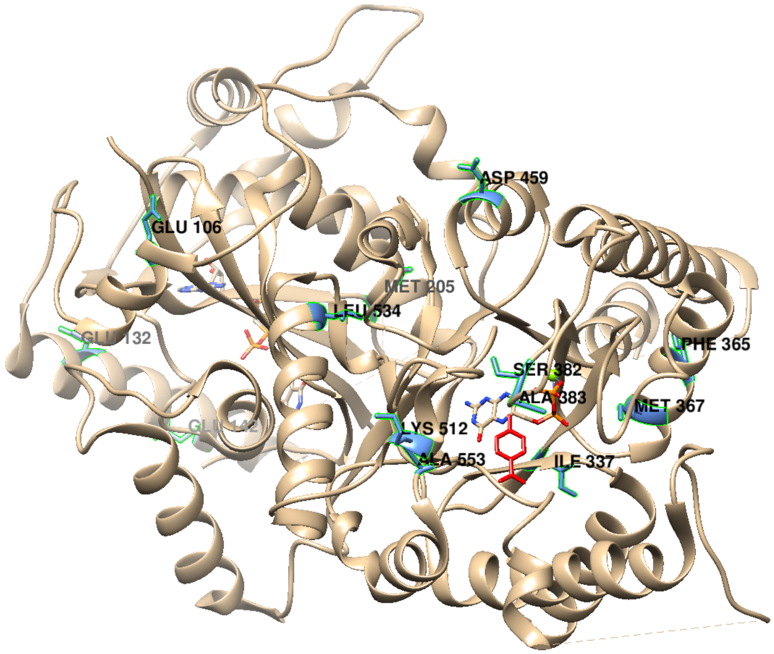

S7 Figure. Amino acid haplotype plot for the MRP1 protein for 496 *P. vivax* isolates from all regions. The tile plot represents amino acid changes in the MRP1 protein for *P. vivax*, and alternative alleles are coloured according to the geographical origin of the isolate. Labels on the right side indicate country of origin (Other EA: Eritrea, Madagascar, Sudan, Uganda; Other SA: Bangladesh, Sri Lanka, Afghanistan; Other SEA: China, Laos, Myanmar; Other SSEA: Indonesia, The Philippines). SNP Positions under the green coloured top band are located within the predicted InterPro domains for the ABC transporter (ATP-binding cassette). Positions under the red coloured top band are located within the ABC transporter (Transmembrane region) InterPro domain. Dashed horizontal lines separate domain regions.

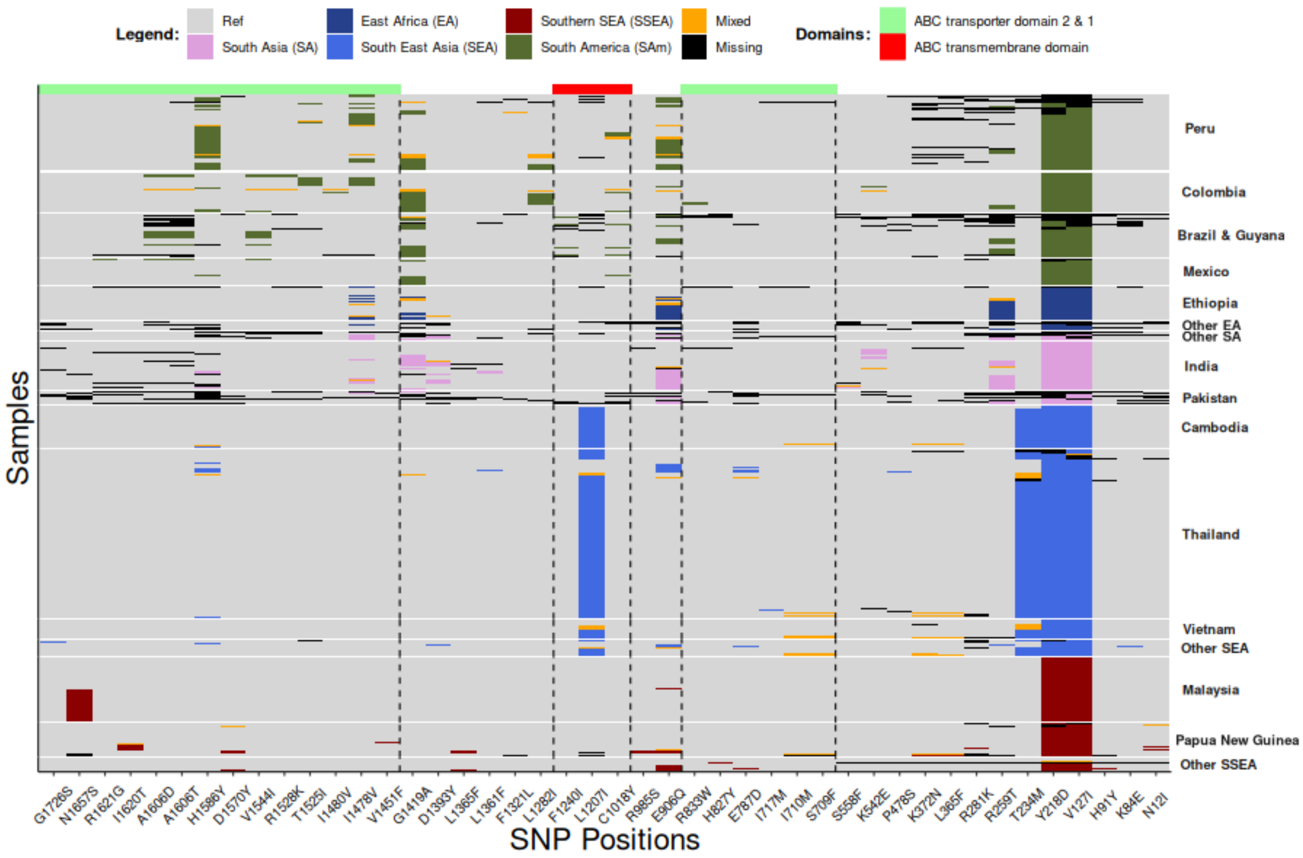

S8 Figure. Genomic regions under selection identified through the integrative haplotype score (*iHS*) applied to *P. vivax* subpopulations from South Asia and East Africa. Manhattan plots showing the genome-wide results of the *iHS* analysis on *P. vivax* subpopulations at different levels: **A)** South Asia region, **B)** Afghanistan, **C)** Pakistan, **D)** India, **E)** East Africa region, **F)** Ethiopia, and **G)** Eritrea. Loci in critical regions (above red lines; *iHS* P< $1x10^{-5}$, two-sided) are reported in **Supplementary Data 6**.

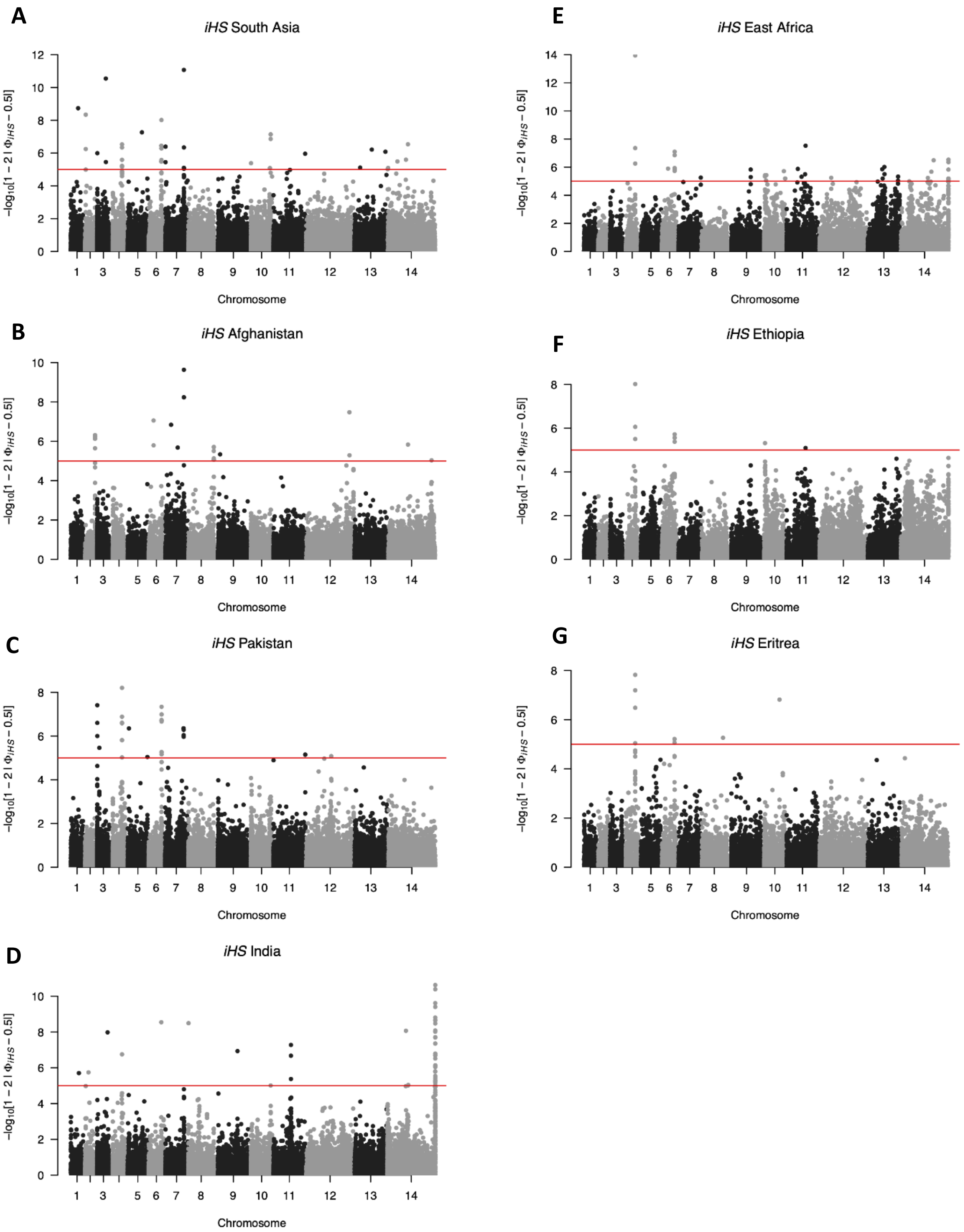

S9 Figure. Genomic regions under positive selection measured through the *Rsb* metric between *P. vivax* subpopulations from South Asia and East Africa. Manhattan plots showing the genome-wide results of the *Rsb* analysis between *P. vivax* subpopulations at different levels. East Africa and South East Asia subpopulations were used as the most geographically proximal subpopulation for comparisons. **A)** South Asia vs. East Africa, **B)** South Asia vs. South East Asia, **C)** Afghanistan vs. Ethiopia, **D)** Afghanistan vs. Thailand, **E)** Pakistan vs. Ethiopia, **F)** Pakistan vs. Thailand, **G)** India vs. Ethiopia, and **H)** India vs. Thailand. Loci in critical regions (above red lines; *Rsb* P< $1x10^{-5}$, two-sided) are reported in **Supplementary Data 7**.

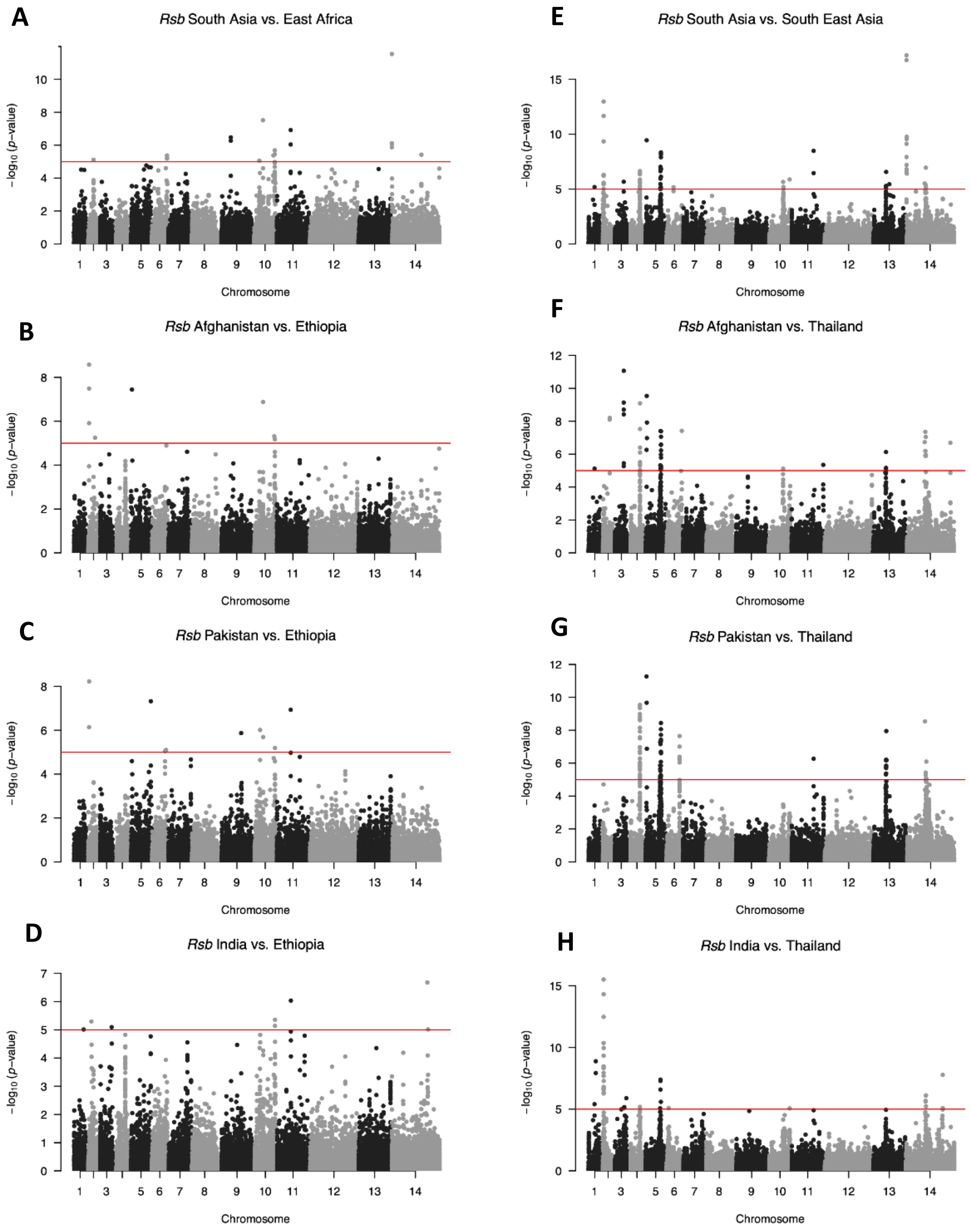

S10 Figure. Genomic candidate regions under positive selection measured through the *Rsb* metric between country-level *P. vivax* subpopulations within the South Asia subpopulation. Manhattan plots showing the genome-wide results of the *Rsb* analysis between *P. vivax* subpopulations at a country level. **(top)** Afghanistan vs. India; **(middle)** Afghanistan vs. Pakistan; **(bottom)** Pakistan vs. India. Loci in critical regions (above red lines; *Rsb* P< 1x10^{-5}, two-sided) with >1 SNP within 10 kbp are reported in **Supplementary Data 8**.

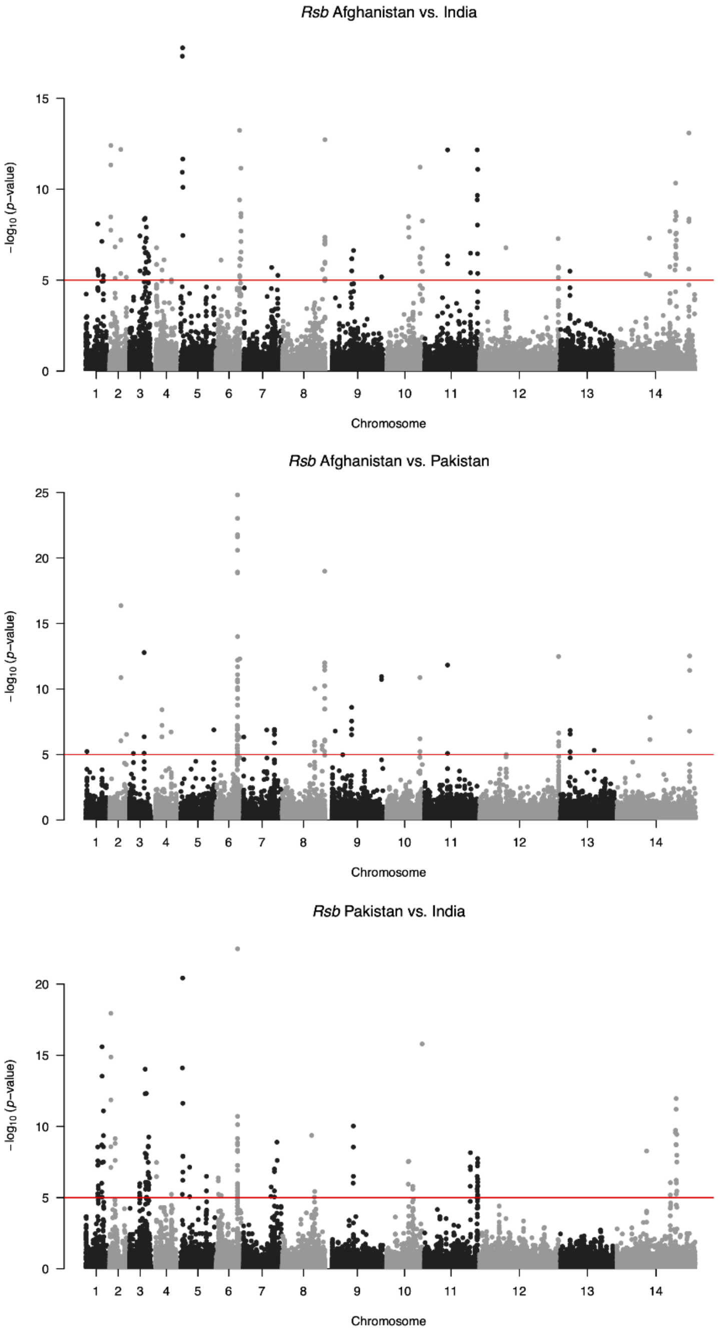

S11 Figure. Boxplots showing the distribution of Tajima's D values in genes with >5 SNPs, by geographical region (A) and country* (B). All boxplots consist of boxes (median and interquartile range) and whiskers that extend to the most extreme data point which is no more than 1.5 times the interquartile range from the box. The analysis includes Afghanistan (20), Pakistan (27), India (28), Ethiopia (22), Cambodia (20), Thailand (84), Papua New Guinea (15), Malaysia (37), Brazil (28), Colombia (31), Peru (44), and Mexico (18).

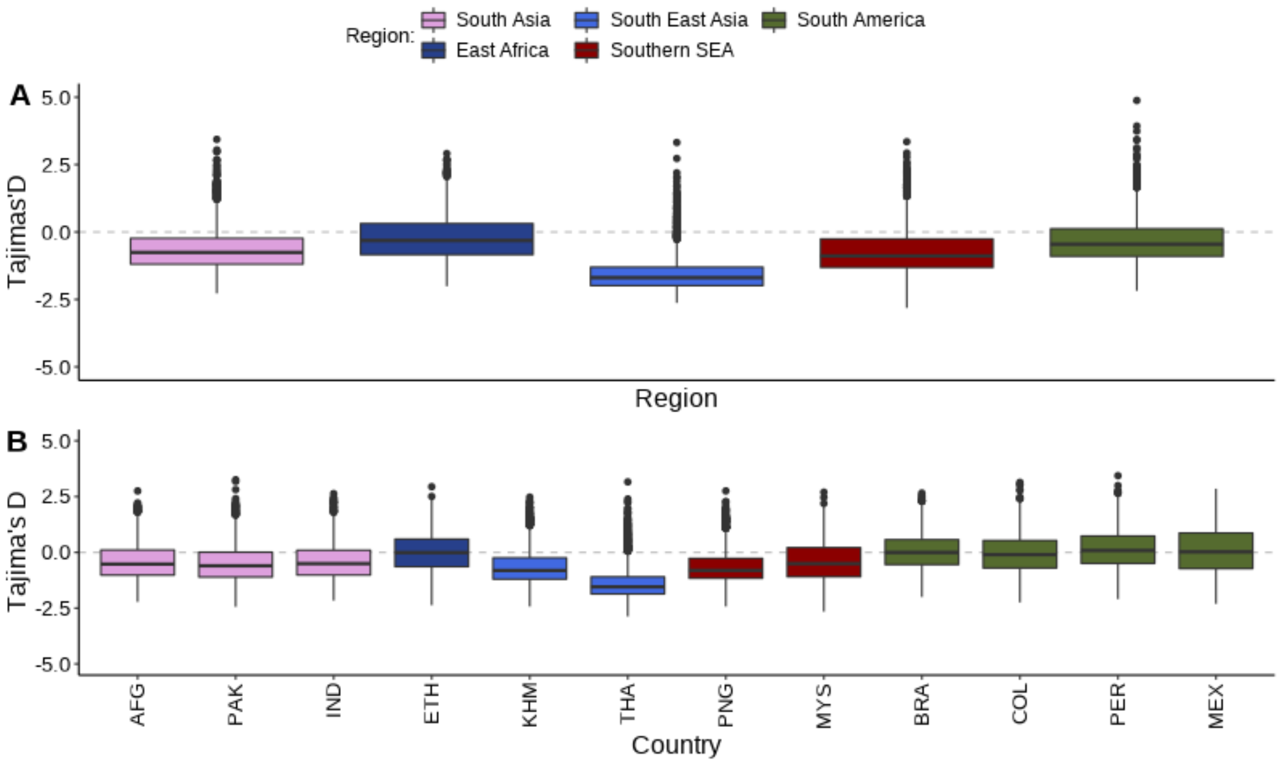

*AFG Afghanistan, PAK Pakistan, IND India, ETH Ethiopia, KHM Cambodia, THA Thailand, PNG Papua New Guinea, MYS Malaysia, BRA Brazil, COL Colombia, PER Peru, MEX Mexico

S12 Figure. Pipeline to process raw sequencing data for *P. vivax.* The pipeline followed the GATK best practice guidelines***.***

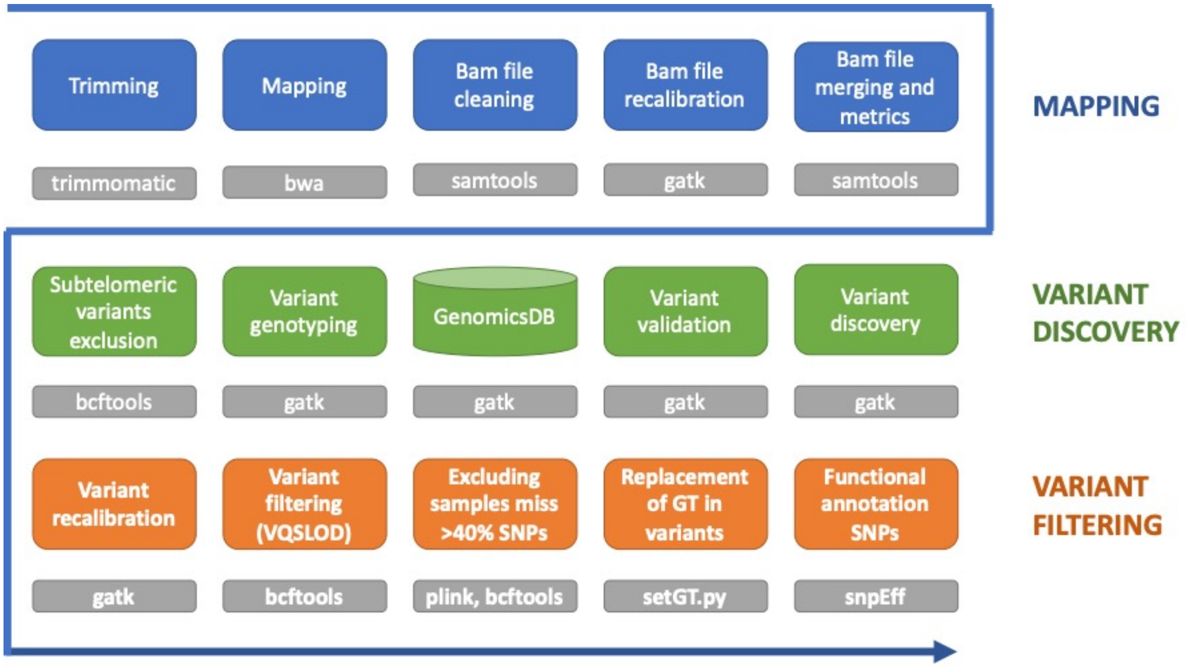

Supplement: Supplementary file 1 — Supplementary Information [file 41467_2021_23422_MOESM1_ESM.pdf]
