# Supplementary material for: Distinctive genetic structure and selection patterns in Plasmodium vivax from South Asia and East Africa
Source: Nat Commun. 2021 May 26;12:3160. doi: 10.1038/s41467-021-23422-3 (PMC8154914; doi:10.1038/s41467-021-23422-3)
Supplement: Supplementary file 2 — Description of Additional Supplementary Files [file 41467_2021_23422_MOESM2_ESM.pdf]

## Description of Additional Supplementary Files

File Name: **Supplementary Data 1**
Description: The samples

File Name: **Supplementary Data 2**
Description: Top 1% of regions with the highest proportion of pairwise Identity-by-descent

File Name: **Supplementary Data 3**
Description: Population differentiation ($F_{ST}$) analysis

File Name: **Supplementary Data 4**
Description: Drug resistance mutations

File Name: **Supplementary Data 5**
Description: *In silico* protein modelling of PVDHFR and PVDHPS

File Name: **Supplementary Data 6**
Description: Positive selection analysis using the integrated haplotype score (*iHS*) in (Southern) South East Asia, South America, South Asia and East Africa

File Name: **Supplementary Data 7**
Description: Positive selection analysis using the between populations (*Rsb*) metric in South Asia and East Africa

File Name: **Supplementary Data 8**
Description: Positive selection analysis using the between countries (*Rsb*) metric in South Asia

File Name: **Supplementary Data 9**
Description: Genes identified in the extreme 0.5 percentiles of Tajima's D values in each country

File Name: **Supplementary Data 10**
Description: Genes with at least one country with a Tajima's D value in excess of 2 (potentially indicative of balancing selection)

File Name: **Supplementary Data 11**
Description: SNPs with high $F_{ST}$ (>0.5; 95%-tile) and *Rsb* (>1.85; 95%-tile) values

File Name: **Supplementary Data 12**
Description: Excluded subtelomeric regions of the PvP01 reference genome
